# Unlocking the Secrets of Extracellular Vesicles: Orchestrating Tumor Microenvironment Dynamics in Metastasis, Drug Resistance, and Immune Evasion

**DOI:** 10.7150/jca.98426

**Published:** 2024-10-14

**Authors:** Rashid Mir, Sadaf Khursheed Baba, Imadeldin Elfaki, Naseh Algehainy, Mohammad A Alanazi, Faisal H Altemani, Faris Jamal Tayeb, Jameel Barnawi, Eram Husain, Ruqaiah I Bedaiwi, Ibrahim Altedlawi Albalawi, Muhanad Alhujaily, Mohammad Muzaffar Mir, Reema Almotairi, Hanan E. Alatwi, Aziz Dhaher Albalawi

**Affiliations:** 1 Department of Medical Laboratory Technology, Prince Fahad Bin Sultan Chair for Biomedical Research, Faculty of Applied Medical Sciences, University of Tabuk, Tabuk, Saudi Arabia.; 2 Watson Crick Center for Molecular Medicine, Islamic University of Science and Technology, J & K, India.; 3 Department of Biochemistry, Faculty of Science, University of Tabuk, Tabuk, Saudi Arabia.; 4 Department of Surgical Oncology, Faculty of Medicine, University of Tabuk, Tabuk, Saudi Arabia.; 5 Department of Clinical Laboratory Sciences, College of Applied Medical Sciences, University of Bisha, Bisha, Saudi Arabia.; 6 Department of Biochemistry, College of Medicine, University of Bisha, Bisha, Saudi Arabia.; 7 Department of Biology, Faculty of Science, University of Tabuk, Tabuk, Saudi Arabia.; 8 King Khalid hospital, MOH, Tabuk 71491, Saudi Arabia.

**Keywords:** Exosomal miRNAs, Extracellular vesicles, tumor microenvironment, epithelial-to-mesenchymal transition, Intercellular Communication

## Abstract

Extracellular vehicles (EVs) are gaining increasing recognition as central contributors to the intricate landscape of the tumor microenvironment (TME). This manuscript provides an extensive examination of the multifaceted roles played by EVs in shaping the TME, with a particular emphasis on their involvement in metastasis, drug resistance, and immune evasion. Metastasis, the process by which cancer cells disseminate to distant sites, remains a formidable challenge in cancer management. EVs, encompassing exosomes and microvesicles, have emerged as critical participants in this cascade of events. They facilitate the epithelial-to-mesenchymal transition (EMT), foster pre-metastatic niche establishment, and enhance the invasive potential of cancer cells. This manuscript delves into the intricate molecular mechanisms underpinning these processes, underscoring the therapeutic potential of targeting EVs to impede metastasis. Drug resistance represents a persistent impediment to successful cancer treatment. EVs are instrumental in intrinsic and acquired drug resistance, acting as mediators of intercellular communication. They ferry molecules like miRNAs and proteins, which confer resistance to conventional chemotherapy and targeted therapies. This manuscript scrutinizes the diverse strategies employed by EVs in propagating drug resistance while also considering innovative approaches involving EV-based drug delivery systems to counteract this phenomenon. Immune evasion is a hallmark of cancer, and EVs are central in sculpting the immunosuppressive milieu of the TME. Tumor-derived EVs thwart immune responses through various mechanisms, including T cell dysfunction induction, the expansion of regulatory T cells (Tregs), and polarization of macrophages towards an immunosuppressive phenotype. In addition, the manuscript explores the diagnostic potential of EVs as biomarkers and their role as therapeutic agents in immune checkpoint blockade therapies. This manuscript provides a comprehensive overview of EV's pivotal role in mediating intricate interactions within the TME, ultimately influencing cancer progression and therapeutic outcomes. A profound understanding of EV-mediated processes in metastasis, drug resistance, and immune evasion opens up promising avenues for developing innovative therapeutic strategies and identifying valuable biomarkers in the ongoing battle against cancer.

## 1. Introduction

Cancer poses a significant threat to human health and is a leading cause of death worldwide 1. Despite extensive research, effective treatments for many types of tumors remain elusive. Clinical tumor therapy faces challenges of limited effectiveness and specificity. Thus, there is an urgent need to develop innovative approaches for precise tumor treatment. Throughout the evolution of molecular medicine, scientists and healthcare professionals have delved into the intricate molecular pathways and mechanisms governing cancer initiation, growth, and advancement 2.

Extracellular Vesicles (EVs) are tiny spherical structures with double-layered membranes, comprised of a wide range of biomolecules such as proteins, nucleic acids, lipids, and metabolites 3, 4, and their composition varies depending on the cell or tissue type of origin 5. Initially believed to be primarily involved in disposing of cellular waste 6, EVs have become vital mediators of intercellular communication, actively engaging in a wide range of normal physiological and pathological processes 7. Their growing recognition stems from their capacity to convey bioactive cargo from one cell to another, facilitating intricate intercellular signaling in healthy or diseased states 4.

A tumor microenvironment (TME) is a complex mixture of cancer cells and an array of non-cancerous cells called cancer-associated stromal cells (CASCs), including immune cells (such as monocytes/macrophages, dendritic cells (DC), neutrophils, natural killer (NK) cells, T cells, and B cells), cancer-associated fibroblasts (CAFs), endothelial cells (EndC), neurons, vasculature, secreted molecules, and the extracellular matrix (ECM) 8-11. Extensive interactions occur between these cells within the TME throughout the stages of cancer development 12. While growth factors, signaling molecules, cytokines, chemokines, and their receptors on both tumor and CASCs facilitate intercellular communication within the TME 13, recently, it has become evident that EVs play a crucial role in intercellular communication in cancers supplying essential nutrients 14, and promoting or inhibiting tumor growth, depending on their cellular origin 1. Both tumor-derived extracellular vesicles (T-EVs) and extracellular vesicles from CASCs (CASCs-EV) reshape their surrounding microenvironment into a supportive ecosystem to promote tumor development, including proliferation invasion, metastasis, angiogenesis, resistance to treatments such as chemo-radiotherapy, targeted therapy, immunotherapy, and anti-angiogenesis therapy across different types of tumors 15-17, immune evasion, pre-metastatic niche formation, and distant metastasis 18-24. Furthermore, although there has been a significant increase in the availability of cancer treatment drugs in recent decades, drug resistance remains a significant hurdle. Cancer patients often respond well to drug therapy initially, but resistance eventually develops and significantly contributes to cancer-related deaths 25. The role of EVs in cancer drug resistance has gained recognition over time. EVs are implicated in drug resistance across various treatment modalities, including radiotherapy, chemotherapy, immunotherapy, targeted therapy, and anti-angiogenesis therapy. Several pathways have been identified as contributors to EV-mediated drug resistance. One mechanism involves drug efflux pumps within EVs, which sequester anticancer drugs, reducing their concentration in cancer cells 26, 27. Specific drug efflux pumps, found abundantly in EVs, can be transferred between cells during chemotherapy, indicating a shared mechanism for conveying drug resistance. Moreover, extracellular vesicles released by drug-resistant cancer cells transport nucleic acids and proteins to susceptible cells, fostering phenomena such as epithelial-mesenchymal transition (EMT), autophagy, metabolic changes, and the emergence of cancer stem cell properties, all associated with acquiring drug resistance 28. EVs can also act as bait, capturing monoclonal antibodies targeting cancer-associated ligands or receptors 28. Moreover, within the TME, cancer cells actively communicate with CASCs through EVs, leading to therapeutic resistance and cancer progression 29. Hence, gaining a more profound insight into the mechanisms of EV-mediated drug resistance has become a pivotal research focus. Cargo within EVs from cancer patients' bodily fluids has been employed as biomarkers for evaluating treatment efficacy. Furthermore, researchers are investigating EVs as a potential target for reversing cancer drug resistance and exploiting their distinctive biological traits as a promising means of delivering therapies to combat resistance.

In this review, we present current insights into the formation, release, and uptake of EVs, focusing on the release of EVs by various cellular components within the TME. We summarize the characteristics of both tumor-derived EVs (T-EVs) and EVs from CASCs and their impact on tumor progression and metastasis within the TME. Specifically, we delve into how EVs facilitate cell-to-cell communication and trigger downstream signaling cascades within the TME, including promoting cancer development. We also incorporate the deregulation of exosomal miRNAs in many cancer types, such as gastric, lung, colorectal, liver, breast, and cervical malignancies. In addition, we investigate the role that EVs play in the development of drug resistance in cancer, as well as the molecular mechanisms that underlie this phenomenon, including the participation of EV-mediated CASCs, miRNAs, lncRNAs, proteins, mRNAs, DNA, and involvement of drug efflux pump in EVs. We also emphasize recent breakthroughs in utilizing EVs to monitor responses to cancer therapy, such as using exosomal miRNAs, lncRNAs, and cirRNAs as biomarkers. Additionally, we discussed EVs potential to tackle drug resistance in cancer treatment via targeting key proteins involved in the biogenesis, secretion, and uptake of EVs and EVs as drug delivery vehicles to overcome drug resistance.

## 2. Role of extracellular vesicles and intercellular communication

**Figure 1:**
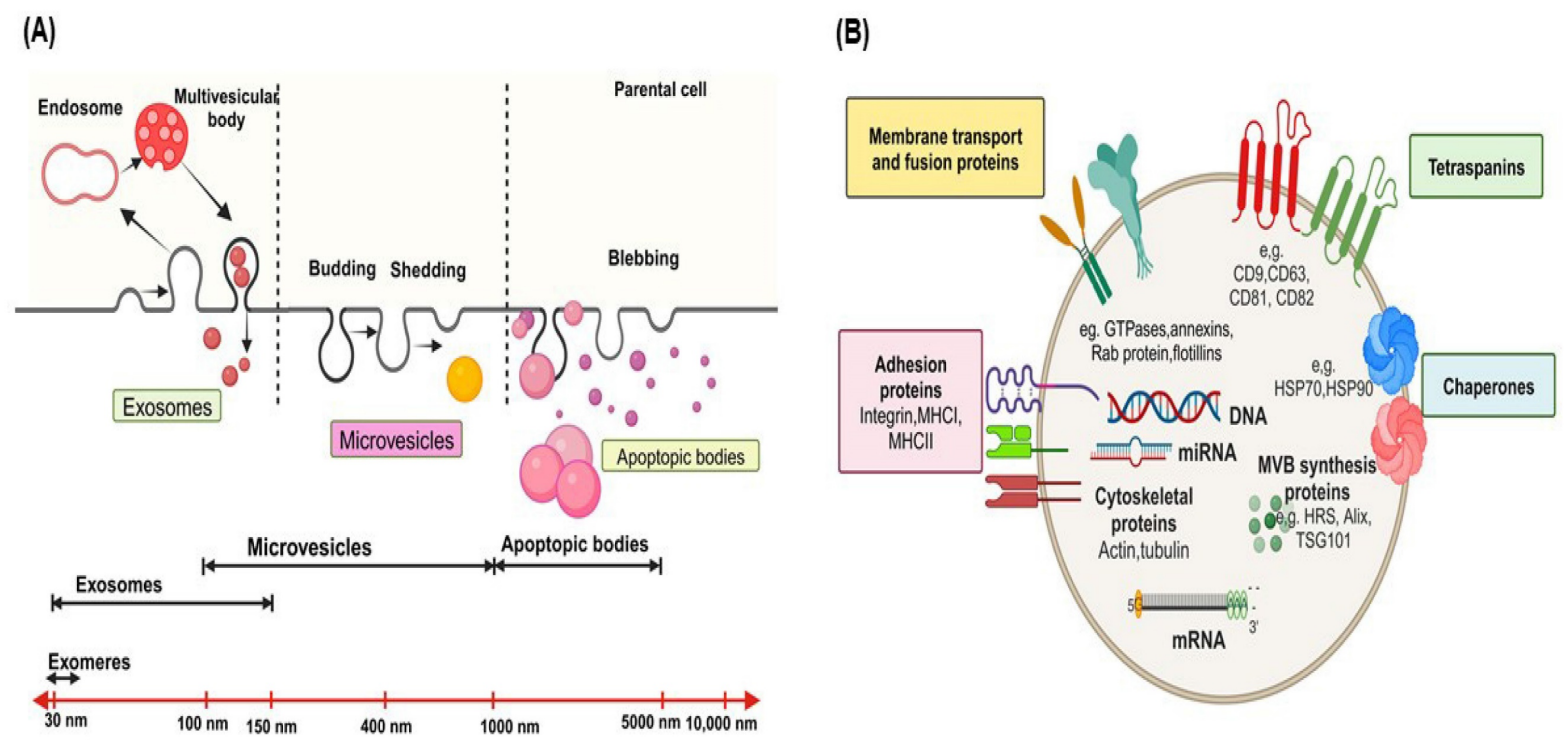
** Biogenesis, Size Ranges and Molecular Composition of Exosomes**. (A) This schematic illustrates the distinct biogenesis pathways of exosomes, microvesicles, and apoptotic bodies. Exosomes originate from the endosomal pathway, where intraluminal vesicles (ILVs) are formed within multivesicular bodies (MVBs) and are eventually released as exosomes upon MVB fusion with the cell membrane. Microvesicles, conversely, are formed through direct outward budding of the plasma membrane, leading to the release of larger vesicles. Apoptotic bodies are generated during programmed cell death (apoptosis) and contain cellular components, including organelles, membrane fragments, and cytoplasmic contents. (B) This section provides information on the characteristic size ranges reported for different vesicle types. Exosomes typically range in size from approximately 30 to 150 nanometers. Additionally, it outlines the molecular composition of exosomes, which includes a diverse cargo of proteins, lipids, nucleic acids (such as miRNA and mRNA), and surface markers, all of which play crucial roles in intercellular communication and modulating various biological processes.

EVs are diverse vesicular structures secreted by various types of cells, either in a continuous or regulated manner, into the extracellular environment 4. These EVs can be found in human biofluids, including blood-derived serum/plasma, urine, saliva, and milk, both in normal physiological conditions and during pathological states 30, 31 and serve as a novel means of intercellular communication between different cell types, facilitating the exchange of biological information and influencing cellular processes. These EVs are classified into three primary subtypes-exosomes, microvesicles (MVs) or microparticles (MPs), and apoptotic bodies-according to their origin and size 3, 32 (**Figure 1A**). While the microvesicles (MVs) arise through cell membrane outward budding and fission 33, exosomes form via fusion between multivesicular bodies (MVBs) containing intraluminal vesicles (ILVs) and the cell's plasma membrane 34 and apoptotic bodies are released during cell apoptosis 29. Exosomes typically measure between 30 to 150 nm, apoptotic bodies range from 1000 to 5000 nm, and microvesicles fall within the size range of 100-1000 nm, sharing characteristics with both exosomes and apoptotic bodies 35.

Recently, even smaller particles known as exomeres, measuring less than 35 nm, have been described, although little is known about their physiological roles, biogenesis, and composition 36. EVs can be classified based on their origin into tumor-derived EVs or mesenchymal stem cell (MSC)-derived EVs (MSC-EVs); their functions into pro-apoptotic EVs or immune-suppressing/stimulating EVs, and based on the presence of specific surface biomarkers, such as CD63^+^, CD9^+^, CD81^+^, or EpCAM^+^ EVs 37.

EVs contain various categories of proteins, such as membrane transport and fusion proteins (**Figure 1B**), including GTPases, annexins, flotillins, and Rab proteins like Rab2, Rab7, Rab11; Tetraspanins like CD9, CD63, CD81, CD82; Chaperones, such as heat-shock proteins (HSP) like HSP70 and HSP90; adhesion proteins like integrins; major histocompatibility complex (MHC I and II) molecules; cytoskeletal proteins like actin, tubulin, and moesin; proteins involved in multivesicular body synthesis, such as HRS, Alix, and tumor susceptibility gene 101 (TSG101); lipid-related proteins 5, 18, 38-42. In addition, proteins like TSG101, Alix, CD9, CD63, CD81, and HSP70 are highly enriched in EVs and commonly used as markers for detecting and purifying EVs 4. EVs contain lipids, including phosphatidylserine, sphingolipids, and cholesterol 43-45. Significantly, phosphatidylserine is uniquely located on the outer surface of the EV membrane, in contrast to its typical position on the inner side of the plasma membrane 44, 46.

The lipid membrane of EVs protects the enclosed nucleic acids, including DNA, mRNA, miRNAs, circular-RNA (circRNAs), and other non-coding RNAs like long non-coding RNAs (lncRNAs) 47, 48, shielding them from degradation by extracellular nucleases 3. These nucleic acids mediate the formation of either a pro-tumoral or anti-tumoral TME, ultimately influencing tumor progression 49. These nucleic acids can serve as identifiers of the donor cells from which the EVs originated and are frequently investigated as disease diagnostic and monitoring biomarkers 50. EVs also contain intact metabolites, such as amino acids, lipids, and intermediates of the tricarboxylic acid (TCA) cycle 51. These metabolites are capable of reprogramming the metabolism of recipient cells. For example, EVs derived from CAFs carry substantial amounts of amino acids and TCA-cycle intermediates. The internalization of these metabolites by nutrient-deprived cancer cells can promote central carbon metabolism, contributing to tumor growth and cancer progression 51. As a result, researchers are exploring the potential of EV metabolites as biomarkers for various diseases 52. In addition, EVs are characterized by unique properties, including highly biocompatible due to their natural origin, the ability to cross biological barriers, low toxicity, and minimal immunogenicity 53. Due to these characteristics, EVs are promising clinical biomarkers and nanocarriers for non-invasive early disease diagnosis, monitoring, and treatment 9, 53, 54.

Following their release from donor cells into the extracellular space, EVs interact with recipient cells and undergo various processes, carrying out their role in intercellular communication upon being taken up by recipient cells 55. It's noteworthy that different types of recipient cells exhibit varying abilities to internalize the same kind of EVs, and the efficiency of EV uptake by the same cell types is also influenced by the source or origin of the EVs. This highlights that the selectivity of EV uptake depends on the specific recipient cell type and the identity of the EVs 56, 57.

Upon contact with recipient cell plasma membranes, EVs are internalized via various endocytic pathways, including micropinocytosis, phagocytosis, lipid raft- or clathrin-caveolae mediated endocytosis 58-61. Surface proteins like integrins, such as integrin-associated protein (IPA) or CD47, are commonly found on EVs and mediate their uptake 62. Other factors involved in EV internalization include β3 integrin 63, heparan sulfate proteoglycans 64, and survivin 65. Once internalized, EVs travel to multivesicular bodies (MVBs) through endocytic routes, where they can either fuse with lysosomes for degradation, supporting recipient cell metabolism, or release/recycle their cargo through endosomes 3. EVs can serve as messengers without being internalized or delivering their contents to recipient cells. For instance, EVs enriched with MHC-II from B lymphocytes can present antigens, initiating antigen-specific T cell responses based on the T cell-activating function of MHC-peptide complexes on EV surfaces rather than their enclosed contents 4.

## 3. Interactions of EV with tumor microenvironment cells

Cancer progression and the development of tumors are influenced by complex and intricate crosstalk between malignant cells and various CASCs within the TME 8, 66. Historically, it was thought that tumor cells directed and instructed CASCs in the TME to adapt and function in ways that support and nourish cancer cells 67. However, recent research has revealed that CASCs can reprogram tumor cells 68, 69, highlighting the bidirectional nature of communication within the TME. These interactions can involve direct cell-to-cell contact and the secretion of signaling molecules that either inhibit or promote tumor progression, depending on the specific signals involved. In the TME, direct cellular junctions, such as cell-cell junctions, are often involved in these interactions. For instance, synaptic connections between neurons and tumor cells in brain tumors enable intercellular signaling, accelerating tumor colonization and progression 70-72. Gap junction proteins, connexins, found between DC cells and tumor cells facilitate the transfer of antigenic peptides from tumor cells to enhance DC-mediated T cell responses, thereby suppressing tumor growth 73. Furthermore, non-cellular components of the TME, including the ECM, cytokines, chemokines, and growth factors, actively participate in intercellular interactions 74, 75. For example, chemokines like CCL2, CCL3, and CCL5 can promote different aspects of tumor development by modulating immune cell activity 76, while others like CXCL8 and CXCL14 mediate more significant anti-tumoral effects 77, 78. The discovery of diverse elements in the TME has dramatically enhanced our comprehension of cancer biology's molecular mechanisms. Furthermore, the growing recognition of EVs in the TME highlights their role as messengers between tumor cells and the microenvironment, significantly influencing local environmental changes 79.

### 3.1 EV-mediated interaction of tumor cells with endothelial cells

**Figure 2:**
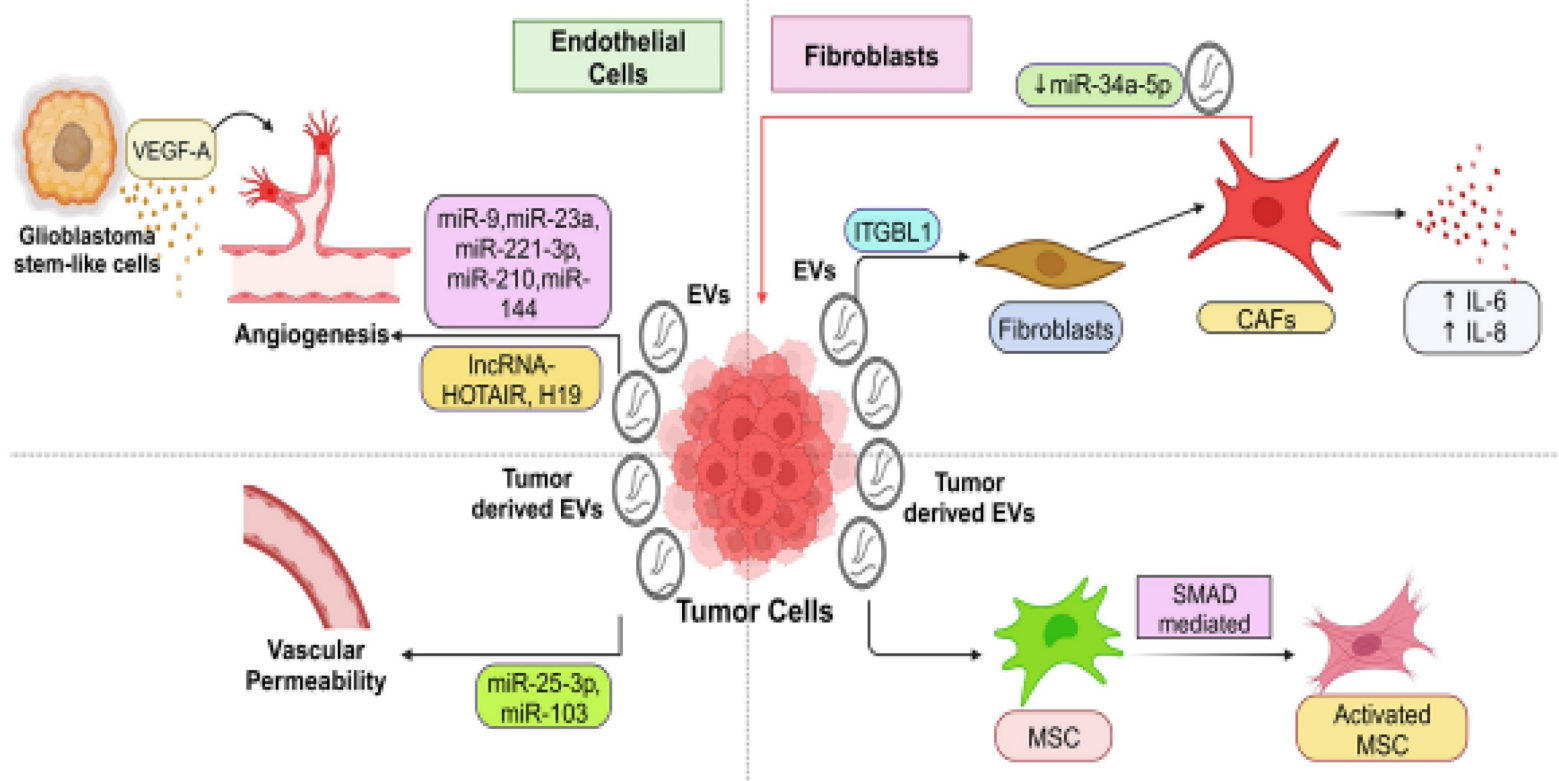
**Extracellular vesicle- mediated crosstalk between tumor cells with endothelial cells and fibroblasts in the TME.** This figure illustrates the intricate cell-cell communication network mediated by EVs within the TME. Tumor and stromal cells, comprising endothelial cells fibroblasts, engage in EV-based signaling that profoundly influences tumor and stromal cell behavior, ultimately creating a favorable TME conducive to tumor growth and progression. EVs originating from tumor cells are shown to be actively involved in communicating with endothelial cells and fibroblasts within the TME. Tumor-derived EVs carry specific cargo, including various bioactive molecules and genetic material, which are transferred to endothelial cells and fibroblasts. This EV-mediated signaling enhances angiogenesis, which promotes the formation of new blood vessels (angiogenesis) that supply nutrients and oxygen to the tumor. Transferring specific molecules can also stimulate fibroblasts to create a supportive matrix that facilitates tumor invasion and metastasis.

T- EVs regulated pro-tumoral functions of endothelial cells (EndCs) are observed in various cancer types, including hepatocellular carcinoma (HCC) 80, nasopharyngeal carcinoma (NPC) 81, 82, glioblastoma (GBM) 83, 84, colorectal cancer (CRC) 85-87, and lung cancer (LC) 88 (**Figure 2**). In EndCs, EVs can be internalized through a process similar to endocytosis and may deliver regulatory biomolecules. Consequently, by secreting EVs, tumor cells can influence EndC functions such as sprouting, branching, and tubular-like structure formation, proliferation, and migration 89, 90. As tumors grow, recruiting new blood vessels is crucial to provide cancer cells with the necessary nutrients and oxygen, a process known as tumor angiogenesis 91, 92. Several growth factors, including fibroblast growth factors (FGF) 93, vascular endothelial growth factor (VEGF) 94, and platelet-derived growth factors (PDGF) 95, play critical roles in regulating tumor angiogenesis. For instance, EVs derived from GBM stem-like cells carry high levels of VEGF-A, inducing angiogenic potential in human brain EndCs 96. EVs containing miRNAs and lncRNAs participate in regulating the pro-tumoral functions of EndCs (**Table 1**). Through the release of ncRNAs, T-EVs can influence angiogenesis. For example, melanoma (MeL)-derived EVs containing miR-9 stimulate EndC migration and tumor angiogenesis by reducing the suppressor of cytokine signaling 5 (SOCS5) levels and activating the JAK/STAT pathway in EndCs 89. Another miRNA, miR-23a, promotes angiogenesis by targeting SIRT1 in recipient EndCs 97. Vesicular miR-221-3P from cervical cancer (CC) inhibits thrombospondin-2 expression in human umbilical vein endothelial cells (HUVECs), enhancing angiogenesis and tumor growth 49. Furthermore, vesicular miR-21-5p from CRC promotes angiogenesis by activating the β-catenin signaling pathway and increasing VEGFA expression 87. MiR-210, abundant in EVs from malignant tumors, stimulates tubular-like structure formation in EndCs, enhancing pro-angiogenic effects and hastening tumor growth. In cases of HCC, the plentiful miR-210 can transfer to HUVECs 80, triggering angiogenesis by reducing SMAD4 and STAT6 expression 80, 98. Additionally, miR-144 plays a pivotal role in angiogenesis in NPC, with vesicular miR-144 suppressing FBXW7 and elevating HIF-1α and VEGFA in recipient cells 82. LncRNAs like HOTAIR and H19 are also involved in angiogenesis. T-EVs carrying lncRNA-HOTAIR induce angiogenesis by upregulating VEGF-A expression in EndCs. LncRNA-H19, closely associated with HCC 99 and liver metastases 100, enhances angiogenesis by upregulating VEGF production 83, 101 in EndCs after being transmitted via T-EVs. Additionally, tumor-derived EVs can increase vascular permeability in endothelial barriers, facilitating cancer cell extravasation and metastasis. For instance, EVs from CRC cells contain miRNA-25-3p, targeting Transcription factor Kruppel-like factors 2 and 4 (KLF2) and KLF4 in EndCs to upregulate vascular permeability 86. Similarly, miRNA-103 transported *via* EVs enhances vascular permeability and promotes metastasis in HCC by targeting transcripts encoding junction proteins 102.

### 3.2 EV-mediated interaction of tumor cells with fibroblasts

T-EVs play a crucial role in inducing the transformation of resident fibroblasts into CAFs (**Figure 2**). CAFs originate from resident fibroblasts, MSCs, and cells undergoing EMT after exposure to tumor-derived EVs 103. CAFs play a central role in shaping the tumor-promoting environment involved in malignant tumor initiation, ECM remodeling, tumor advancement, and metastasis 104, 105. EVs originating from Hodgkin lymphoma can transform healthy fibroblasts into disease-associated CAFs by initiating the NF-κB signaling pathway, producing neo-angiogenic factors 106. EVs containing integrin beta-like 1 (ITGBL1) from CRC cells trigger the transformation of local fibroblasts into CAFs through the TNFAIP3-mediated NF-κB signaling pathway. These activated CAFs release various pro-inflammatory cytokines, such as IL-6 and IL-8, which facilitate the creation of pre-metastatic sites and support the metastatic process 107. Several studies have highlighted the importance of functional biomolecule delivery in regulating the transformation of CAFs 108-112. For example, EVs originating from chronic lymphocytic leukemia (CLL) cells prompt the conversion of fibroblasts into CAFs by transferring regulatory proteins and miRNAs from the parent cells, ultimately accelerating tumor growth. 108. It's important to note that MSCs and resident fibroblasts EV can also differentiate into an activated, myofibroblast-like phenotype, contributing to tumor progression within the TME 113. EVs derived from BC, ovarian cancer (OVC), and gastric cancer (GC) cells have demonstrated the ability to convert adipose tissue-derived MSCs into an activated, myofibroblast-like state with pro-tumor functions, frequently involving the SMAD-mediated signaling pathway 114-116. However, the specific EV cargoes and underlying molecular mechanisms involved in these processes require further investigation. Furthermore, fibroblasts, including CAFs, can signal cancer cells via EVs once activated. They transport lipids and proteins from CAFs to nearby cells, including cancer cells, increasing tumor growth 117. Enhanced migration and invasion of cancer cells following education by CAF-derived EVs are attributed to their tumor-promoting actions 118. One study revealed that CAF-derived EVs contain lower levels of miRNA-34a-5p compared to EVs from normal fibroblasts. MiRNA-34a-5p can directly target AXL, limiting cancer cell proliferation and progression. Deleting miRNA-34a-5p in CAF-derived EVs increased AXL expression in recipient cancer cells, activating the AKT/GSK-3/β-catenin signaling cascade and promoting EMT and metastasis 119. Most research indicates a reinforcing loop where cancer cells and fibroblasts mutually enhance their support through EV-mediated communication, ultimately driving tumor advancement. Disrupting these EV-mediated interactions between cancer cells and fibroblasts could potentially serve as a therapeutic approach.

### 3.3 EV-mediated interaction between tumor cells and immune microenvironment

**Figure 3:**
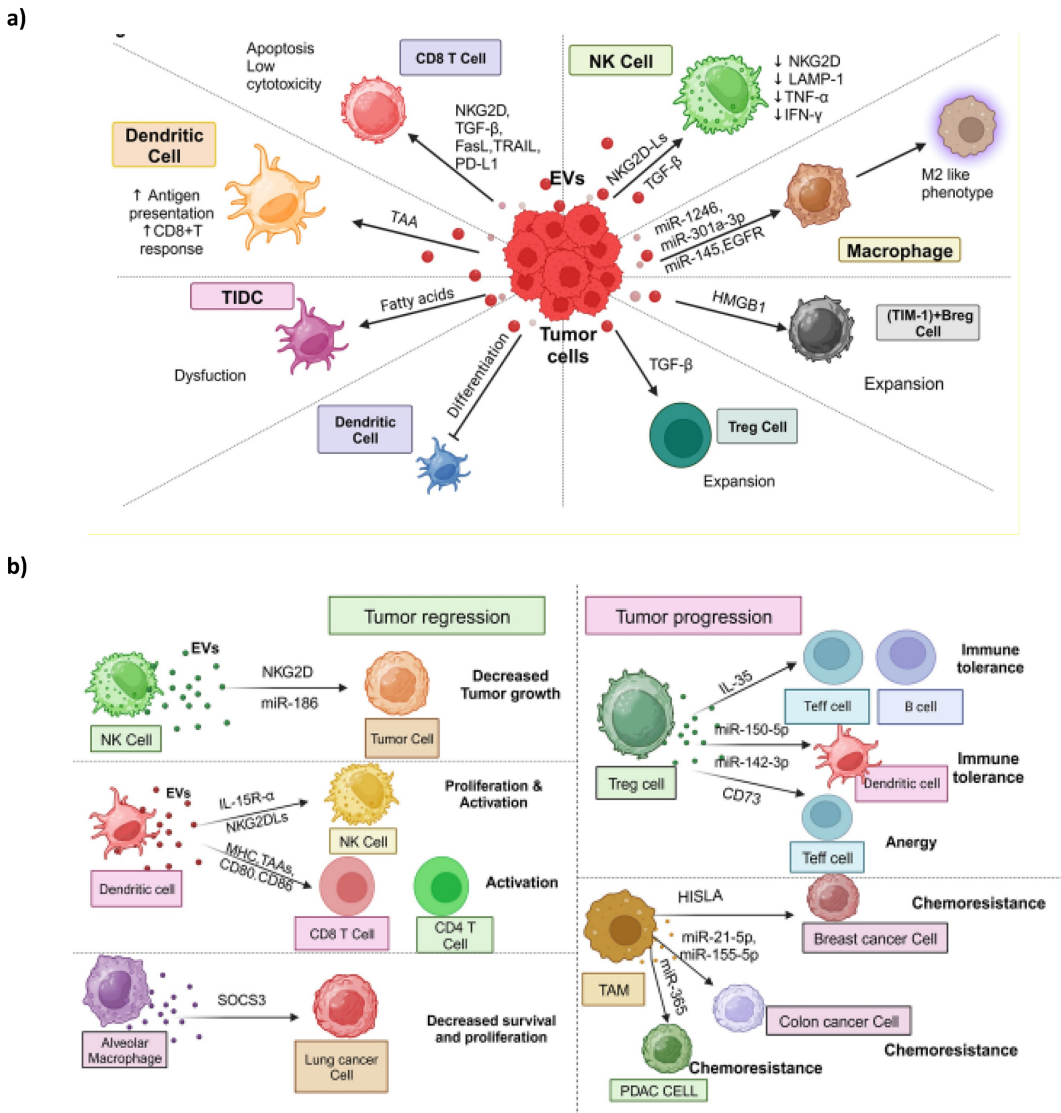
**Extracellular vesicle- mediated crosstalk between tumor cells and Immune microenvironment.** This diagram shows the complex web of cell-to-cell communication inside the TME that EVs facilitate. Both tumor and immune cells participate in EV-based signaling, which has a significant impact on the behavior of both types of cells, thereby contributing to TME (**A**) **Tumor-Derived EVs' Role in the Tumor Immune Microenvironment:** the pivotal role of tumor-derived EVs in shaping the Tumor Immune Microenvironment (TIME) is depicted. Tumor cells release EVs that contain various signaling molecules, such as cytokines, chemokines, and antigens. These EVs interact with immune cells, including T, B, and myeloid cells, within the TME. Tumor-derived EVs can have immunosuppressive effects, dampening the immune response and promoting immune evasion by inhibiting the activation of cytotoxic T cells and promoting the expansion of regulatory T cells (Tregs). Conversely, some EVs can have pro-inflammatory effects, further fuelling the immune response and contributing to chronic inflammation within the TME. **(B) The role of immune cells-derived extracellular vesicles is to play a dichotomous role in tumor formation.** This figure illustrates the dual roles of immune cell-derived EVs in the tumor microenvironment. On the left side, NK cell-derived EVs containing miR-186 suppress neuroblastoma tumorigenic growth. Dendritic cell (DC)-derived EVs possess IL-15Rα and NKG2D ligands, promoting NK cell proliferation and activation. DC-derived EVs carrying MHC molecules, tumor-associated antigens, and costimulatory molecules activate CD8+ and CD4+ T cells. Alveolar macrophage-derived EVs transfer suppressors of cytokine signaling 3 (SOCS3), repressing the survival and proliferation of lung cancer cells. On the right side, immune-suppressive cell-derived EVs exhibit pro-tumoral effects. Treg cell-derived exosomes loaded with IL-35 propagate immune tolerance through effects on effector T cells and B cells. miR-150-5p and miR-142-3p from Treg cells via EVs induce a tolerogenic phenotype in DCs. Treg cell-derived exosomes carrying CD73 induce effector T cell anergy. EVs derived from tumor-associated macrophages (TAM) containing miRNAs or lncRNAs promote tumor cell migration, invasion, and chemoresistance. For example, TAM-derived EVs containing lncRNA HISLA induce chemoresistance in breast cancer cells. This figure highlights immune cell-derived EVs' complex and dualistic nature in promoting antitumor immune responses and driving tumor progression.

Within the TME, immune cells play a crucial role in detecting and responding to cancer cells 120. However, cancer cells can manipulate signaling pathways within these immune cells, rendering them immunosuppressive and promoting tumor cell survival and proliferation 121. Emerging evidence suggests that T-EVs are essential signaling molecules contributing to abnormal immune responses in the TME. While specific subsets of T-EVs have immune-stimulatory effects targeting immune cells in the tumor immune microenvironment (TIME) 18, 122-124, they are generally involved in reshaping the TIME (**Figure 3a).** In addition, we discussed the dual role of immune cell-derived EVs in the tumor microenvironment; some are involved in tumor regression, and others in tumor progression (**Figure 3b**).

#### 3.3.1 Involving NK cells

NK cells are among the first responders in detecting and responding to cancer cells. NK cells express the receptor NK^G2D^, which allows them to recognize stress-associated molecules on the surface of cancer cells, aiding in cancer cell detection 125. Studies have shown that EVs derived from PCa cells can inhibit NK cell activity. These EVs carry NK^G2D^ ligands on their surface, leading to the downregulation of NK^G2D^ on NK cells and a reduction in NK cell cytotoxicity 126. Additionally, T-EVs can downregulate the levels of lysosome-associated membrane protein 1(LAMP1), tumor necrosis alpha (TNF-α), and INF-γ, collectively attenuating NK cell cytotoxicity and inhibiting the expression of CD71 and CD98, thereby limiting glucose uptake in NK cells 151 (**Figure 3a**). Moreover, EVs derived from pancreatic cancer (PC) cells are rich in TGF-β1, which can hinder the expression of CD107a and IFN-γ in NK cells. Mechanistically, TGF-β1-carrying EVs activate the TGF β-Smad2/3 pathway in NK cells, further impairing NK cell-mediated cytotoxicity 127. Conversely, NK cells release EVs that contain functional molecules like NK^G2D^/CD94, perforin, and granzymes, contributing to NK cell cytotoxicity and presenting a potential therapeutic approach to enhance NK cell anti-cancer actions 128 (**Figure 3b**). For instance, EVs derived from NK cells carry functional miR-186, which can target genes such as v-Myc avian myelocytomatosis viral oncogene neuroblastoma derived homolog (MYCN), Aurora-A kinase (AURKA), transforming growth factor-beta (TGF-β) receptor type 1 (TGFBR1), and transforming growth factor-beta (TGF-β) receptor type 2 (TGFBR2) in neuroblastoma or NK cells. This inhibition suppresses neuroblastoma's tumorigenic potential and prevents TGF-β-dependent inhibition of NK cells 129.

#### 3.3.2 Involving dendritic cells

DCs are crucial antigen-presenting cells coordinating immune responses (**Figure 3a**). T-EVs carry and transfer tumor antigens to DCs, leading to robust CD8**^+^** T cell-dependent anti-tumor immune responses 130. Additionally, EVs derived from macrophages transfer antigens to DCs, promoting CD4**^+^** T-cell responses 131. In these scenarios, EVs typically enhance anti-tumor activity by facilitating antigen presentation. However, within the TME, DC function can be compromised due to various immunosuppressive factors 132. For example, T-EVs containing fatty acids can induce metabolic changes in tumor-infiltrating dendritic cells (TIDCs), causing immune dysfunction by promoting fatty acid oxidation and inhibiting glucose uptake by TIDCs 133. Furthermore, several studies have demonstrated that tumor-derived EVs can inhibit the differentiation of DCs from myeloid precursors in the bone marrow 134 and from monocytes 135 while promoting the expansion of myeloid-derived suppressor cells (MDSCs) 135, 136. Nonetheless, it's important to note that not all EV uptake by DCs leads to enhanced immunity. Some research has found that EVs derived from T cells can down-regulate peptide/MHC antigen I and induce apoptosis in DCs, thus suppressing the CD8**^+^** cytotoxic T cell response 137.

On the other hand, EVs derived from DCs have been shown to stimulate immune responses 138 (**Figure 3b**). These DC-derived EVs may carry NK^G2D^Ls (NK^G2D^ ligands) and functional IL-15 receptor alpha (IL-15 Rα), promoting NK cell proliferation and activation, ultimately resulting in anti-metastatic effects mediated by NK1.1+ cells 139. Moreover, DC-derived EVs containing MHC I and II, T cell costimulatory molecules, and tumor-associated antigens can prime cytotoxic T lymphocytes (CTLs) *in vivo* and suppress the growth of established murine tumors 140. Similarly, exosomes derived from DCs expressing antigens from hepatocellular carcinoma (HCC) induce an effective antigen-specific immune response and reshape the tumor microenvironment from immunoinhibitory to immuno-stimulatory, leading to tumor shrinkage 141. Additionally, a study demonstrated that EVs derived from activated DCs via TLR 4 signaling stimulated macrophages and DCs, resulting in stronger anti-tumor immunity *in vivo* 142. These findings suggest that DC-derived EVs hold potential utility for cancer immunotherapy.

#### 3.3.3 Involving macrophages

Macrophages are a notable immune cell population in the TME, playing a crucial role in immune-oncological dynamics, often engaging in significant interactions through EVs. In the TME, tumor-associated macrophages (TAMs) primarily exhibit a tumor-promoting M2-like phenotype characterized by immunosuppression and the facilitation of tumor growth and advancement 143. Macrophage polarization, i.e., conversion of antitumor M1 subtype to M2 subtype, is influenced by various factors, and tumor-derived EVs containing functional components like miRNA, lncRNAs, and proteins have emerged as critical regulators of macrophage polarization (**Figure 3a**). For example, EVs from epithelial OVC can transfer various miRNAs to macrophages, inducing an M2 phenotype, especially in the hypoxic TME 144. Hypoxic EVs from PC and GBM induce M2-like macrophages 145, 146. In GBM-derived EVs, an increased content of miR-1246 plays a role in inducing M2 macrophage polarization by activating the STAT3 signaling pathway, suppressing the NF-κB signaling pathway 146, and elevating TGF-β activity 147. Hypoxic EVs from PC cells are enriched with miR-301a-3p, which down-regulates PTEN expression and activates the PI3Kγ pathway, increasing CD163 expression, a marker of M2 macrophages 145. The M2 macrophage phenotype creates a TIME and promotes tumor cell migration and invasion. EVs from CRC cells have transferred miR-145, resulting in the down-regulation of histone deacetylase 11 in macrophages and subsequent M2 polarization 148. Macrophage-derived EVs, on the other hand, have received less attention regarding their effects on other cells but are potentially significant (**Figure 3b**). For example, EVs from TAMs can enhance glycolysis and chemoresistance in BC cells by delivering HIF-1α-stabilizing lncRNA (HISLA) 149. The transfer of exosomal miR-365 from TAMs can influence pyrimidine metabolism and cytidine deaminase expression in pancreatic cancer (PC) cells, ultimately leading to chemotherapy resistance 150.

Additionally, EVs originating from M2 macrophages have demonstrated the ability to enhance the invasion and migration of CRC cells by transmitting miR-155-5p and miR-21-5p 151. Interestingly, in certain cases, macrophage-derived EVs may exert anti-tumor effects. For instance, EVs from alveolar macrophages (AMs) hinder the proliferation and survival of LC cells by delivering suppressors of cytokine signaling 3 (SOCS3) 152. Further investigation is required to fully comprehend the multifaceted role of macrophage-derived EVs in the TME.

#### 3.3.4 Involving T-cells

T cells have pivotal and diverse roles in cancer immunology. Cytotoxic T cells and helper T cells are at the forefront of tumor immunity, while T regulatory cells (Tregs) contribute to the evasion of the immune response. Within the TME, T-EVs act as immune suppressors, fostering a pro-tumor environment by promoting the recruitment and activation of Tregs 49 (**Figure 3a**). Notably, T-EVs containing transforming growth factor-beta (TGF-β) play a role in expanding Tregs from peripheral blood precursors, and these developed Tregs can, in turn, produce TGF-β to exert immunosuppressive effects 153, 154. In certain instances, such as in NPC, T-EVs recruit Tregs to the TME and transform T cells into Tregs, intensifying immunosuppression 155. The up-regulation of CCL20 transcription by NPC cells attracts Tregs 155. Cytotoxic T cells are pivotal for targeting cancer cells by detecting tumor-associated antigens on cancer cells and inducing apoptosis in them through the secretion of granzymes and perforins 156, 157. However, T-EVs can hinder the function of CD8**^+^** cytotoxic T cells by down-regulating the expression of NK^G2D^, thereby facilitating immune evasion 126. Moreover, exosomes released by colorectal cancer (CRC) cells, carrying Fas ligand (FasL) and TNF-related apoptosis-inducing ligand (TRAIL), can trigger apoptosis in activated CD8^+^ T cells, further promoting immune escape 158. Tumor cells often evade immune surveillance by engaging inhibitory immune checkpoint signaling through the interaction of programmed death-ligand 1 (PD-L1) on their surface with programmed death-1 (PD-1) on effector T cells. T-EVs carry PD-L1 on their surface, which suppresses the function of CD8+ T cells, aiding immune evasion 159, 160. Immunosuppressive cells release EVs with immunosuppressive functions (**Figure 3b**). For instance, Treg cell-derived EVs bear IL-35 on their surface, inducing an immunosuppressive phenotype in recipient T and B cells, resulting in secondary immune suppression 161. Additionally, Treg cell-derived exosomes containing CD73 can convert extracellular adenosine-5-monophosphate (AMP) into adenosine, inhibiting the production of IL-2 and interferon-gamma (IFN-γ), thereby suppressing the function of effector T cells 162. Several miRNAs also contribute to Treg cell-derived EV-mediated immunosuppression, such as miR-150-5p and miR-142-3p, which induce a tolerogenic phenotype in DCs 163. Although less extensively studied in this context, B cells 164 and neutrophils 164 also partake in EV-mediated interactions within the TIME. Tumor-derived EVs can modulate regulatory B (Breg) cells, expanding TIM-1**^+^** Breg cells that secrete IL-10 and impair CD8**^+^** T cell functions 165. Regarding neutrophils, reports have demonstrated that T-EVs can influence neutrophils to support tumor progression 166-168.

## 4. Exosomal-miRNAs' and their functions in various carcinomas

There has been recent speculation that tumoral cells produce excessive exosomes. Exosomes are an important group of extracellular vesicles that help with cell-to-cell contact and the transfer of various biomolecules. Recipient cells may be exposed to miRNAs via exosomes, which then form a RISC complex that can either degrade the target mRNAs or prevent the translation of the associated proteins. As a result, miRNAs produced from exosomes play a significant role in recipient cell gene regulation. It may impede tumor development, invasion, metastasis, angiogenesis, and treatment resistance by interfering with the microenvironment and tumor immunity. Consequently, exosomal miRNAs play a crucial role in controlling the advancement of cancer. Exosome composition, in particular the quantity of microRNAs (miRNAs) within these vesicles, exhibits a pattern unique to each disease that reflects pathogenic processes and can be used as a predictive and diagnostic indicator 169. It is documented that exosome-mediated delivery of miRNAs are found to be linked to the etiology of lung, gastrointestinal, breast and cervical carcinoma (**Table 2**).

Exosomal microRNAs, depending on whether they are upregulated or downregulated, play critical roles in either the development or prevention of NSCLC through a variety of signaling pathways. For instance, Exosomal miR-3180- 3p can stop lung cancer cells from proliferating and from being able to spread by inhibiting the expression of FOXP4 170. Similar to this, miR-770 transported via exosomes limits M2 macrophage polarization by affecting MAP3K1 expression. This would lessen the lung cancer cells' capacity for invasion 171. In addition, exosome-transferred miR-338-3p is found to inhibit CHL1 via the MAPK signaling pathway, which in turn suppresses lung cancer metastasis 172. Conversely, lung cancer-originated exosomal miR-3157-3p has also been shown to upregulate vascular permeability and to increase angiogenic and metastatic potential 173. Moreover, exosomal miR-1260b generated from lung cancer has been demonstrated to upregulate these cells' capacity for metastasis by inhibiting HIPK2 expression 174. Furthermore, this kind of cancer may spread more easily if tumor-associated macrophages secrete miR-155 and miR-196a-5p in their exosomes 175.

Exosome-mediated delivery of miRNAs is also implicated in the etiology of gastro-intestinal carcinoma. For instance, it has been shown that in patients with gastric cancer, there is a corelation between exosomal miR-590-5p down-regulation and a low overall survival rate. Studies conducted *in vitro* have demonstrated that over-expression of miR-590-5p inhibits the migration and invasion of cells in gastric cancer cells 176. Another downregulated miRNA found in serum-derived exosomes from individuals with this kind of malignancy is miR-122-5p. By suppressing GIT1 expression, exosome-mediated transport of miR-122-5p may limit the ability of gastric cancer cells to proliferate and spread 177. In contrast to early-stage samples, tissues and serum exosomes from advanced stages of gastric cancer have been found to have an overexpression of miR-10b-5p. miR-10b-5p could target KLF11 in fibroblasts and PTEN in stomach cancer cells. The suppression of miR-10b-5p increases PTEN levels and represses PI3K/AKT/mTORC1 signaling in gastric cancer cells, which lowers the cells' capacity to form colonies and their viability 178. Numerous miRNAs that have been found to influence the growth of colorectal cancer have been found in exosomes produced from cancer. For example, miR-21-5p transferred from colorectal cancer cells to endothelial cells via exosomes suppressed KRIT1 expression in recipient HUVEC cells. This, in turn, induces the activity of β-catenin signals and VEGF-A and Ccnd1 expression. In their entirety, these modifications in expression promote angiogenesis and increase vascular permeability. Additionally, it has been demonstrated that patients with colorectal cancer have higher quantities of this miRNA in circulating exosomes than do healthy donors 87. An analysis of serum exosomal miR-92a-3p and miR-17-5p for detecting primary and metastatic colorectal cancer (CRC) revealed a direct correlation between the stages of CRC17 and the up-regulation of these miRNAs 179. Another study found that in colon cancer patients at stages II and III, low expression of circulating exosomal miR-4772-3p, isolated from serum, had been thought to be a predictive biomarker for cancer recurrence 180. In hepatocellular carcinoma, reduced expression of VE-cadherin and ZO-1 in endothelial cells has demonstrated the predictive usefulness of exosomal levels miR-638 in serum samples 181. Conversely, mesenchymal stem cells have been demonstrated to secrete exosomal miR-15a, which can suppress SALL4 levels and obstruct the advancement of this particular cancer 182.

Furthermore, exosome-derived miRNAs also have the potential to impact the etiology of breast and cervical cancers. One of the leading causes of breast cancer patients' poor overall survival rates is metastasis. Exosomal miRNAs are essential for nearly all stages of numerous biological processes related to breast cancer 183. In various investigations, exosomal miRNAs have been shown to have a dual function in breast cancer metastasis and associated processes. Within the framework of cervical cancer, cancer cells produce exosomes containing miR-1468-5p, which causes immunological escape by converting lymphatic capillaries to immunosuppressive 184. Furthermore, by preventing the production of vinculin in vascular endothelial cells, the delivery of miR-663b by these exosomes increases the angiogenic potential of cervical cancer cells 185.

## 5. Extracellular vesicles from TME cells induce therapeutic resistance in cancers

Drug resistance continues to be a significant obstacle to successful cancer treatment, contributing to around 90% of cancer-related fatalities. EVs induce resistance to therapy by sequestering or removing drugs from cancer cells and regulating signaling pathways associated with processes like EMT, autophagy, metabolism, and the maintenance of cancer stemness 29. This section focuses on the contribution of EV-mediated miRNAs and lncRNAs to drug resistance **Table 3**.

### 5.1 Resistance due to EV-mediated miRNAs

miRNAs are frequently dysregulated in cancer and are implicated in drug resistance 186, 187. When EVs transfer miRNAs from drug-resistant cancer cells to drug-sensitive ones, they alter gene expression in the recipient cells, making them more resistant to drugs. Examples include miR-221 and miR-222 from tamoxifen-resistant BC cells 188, miR-210 from gemcitabine (GeM)-resistant PC stem cells 189, miR-500a-3p from cisplatin-resistant GC cells 190, miR-31-5P from sorafenib resistant renal cell carcinoma (RCC) 191, miR-222-3P from GeM resistant non-small cell lung cancer (NSCLC) cells 192, miR-761 from pazopanib resistant synovial sarcoma (SS) 193, miR-46146 from oxaliplatin resistant CRCs 193, miR-21 from 5-fluorouracil (5-FU) resistant CRCs 194, and miR-21 from cisplatin-resistant OSCC 195. These miRNAs can target genes associated with drug response, leading to drug resistance.

Moreover, miRNAs carried by EVs play a significant role in fostering multidrug resistance (MDR) in acute myeloid leukemia (AML) cells. For example, miR-19b and miR-20a display distinct expression patterns between AML cells that responded to chemotherapy and those that developed chemoresistance 196. Additionally, BC cells release a variety of EV miRNAs following chemotherapy, including miR-203a-3p, miR-195-5p, and miR-9-5p. These specific miRNAs target the transcription factor ONECUT2 197, leading to the emergence of cancer stem cell-like characteristics and chemotherapy resistance. EVs obtained from docetaxel-resistant prostate cancer (PCa) cell lines contain an abundance of miR-598, miR-34-a, miR-148a, miR-146a, and miR-34. Among these, miR-34 plays a regulatory role in BCL-2, potentially impacting apoptosis in response to drug treatment 198. Similarly, higher miR-21-5p, miR-1246, miR-1229-5p, and miR-96-5p levels were detected in drug-resistant CRC cells and the EVs of chemoresistant patients compared to their sensitive counterparts. The transmission of these miRNAs via EVs contributes to the development of drug resistance across various cancer types, presenting both challenges and opportunities for comprehending and surmounting drug resistance in cancer therapy 199. In addition to chemotherapeutic resistance, miRNA in EVs plays diverse roles in radiotherapy resistance (RR). For instance, miR-208a is upregulated in the serum of LC patients after radiotherapy 200. Exosomes transport it and induce RR by targeting p21 and activating LC cells' Akt/mTOR pathway. Another study identified miR-603 as the most altered miRNA in GBM samples before and after radiation treatment. Ionizing radiation triggers the release of miR-603 via EVs, reducing IGF1 and IGF1R expression, promoting a cancer stem-cell state, enhancing DNA repair, and leading to RR in GBM 201.

### 5.2 Resistance due to EV-mediated lncRNAs

EVs deliver lncRNAs to recipient cells, modulating their response to drugs. Examples include lncRNA HOTAIR in EVs from GBM patients conferring resistance to TMZ lncRNA NEAT1 from BC, promoting resistance to cisplatin, PTX, and 5FU. Drug-resistant ALK translocated LC cells secrete lncRNAs MEG3 and XIST in EVs, inducing drug resistance in other cell subpopulations and maintaining intratumoral heterogeneity 202. The transmission of EVs carrying lncRNAs like SNHG14 in BC contributes to drug resistance by activating the Bcl-2/Bax signaling pathway while inhibiting apoptosis 203. Similarly, EV-mediated transfer of lncRNA RP11-838N2.4 may promote erlotinib resistance in NSCLC cells 204. In HCC, EVs contain lncRNAs such as VLDR and ROR, contributing to drug resistance 205, 206. Additionally, EVs transmit linc-VLDLR in esophageal cancer (EC) cells, promoting drug resistance by upregulating ABCG2 expression 207. EVs from HCC cells also enrich lncRNA ROR, reducing chemotherapy-induced cell death 190. Moreover, lncRNA H19 delivered via exosomes promotes resistance to gefitinib in NSCLC cells 208, erlotinib resistance in NSCLC 209, and doxorubicin resistance in BC 210. Exosomes released by NSCLC cells transport lncRNA SOX2 overlapping transcript (SOX2-OT) to macrophages, promoting M2 polarization and enhancing EGFR-TKI resistance in NSCLC cells 211.

### 5.3 Resistance due to EV-mediated proteins

EVs also carry functional proteins that play a role in developing drug resistance in cancer cells (**Table 3**). One study suggests that CRC cells acquire resistance to 5-FU by transferring phosphorylated STAT3 (signal transducer and activator of transcription 3) via exosomes 212. Another study demonstrates that intercellular transfer of TrpC5 (transient receptor potential channel 5) through EVs enables recipient cells to acquire Ca^2+^-permeable channels, leading to increased production of P-glycoprotein (P-gp/ABCB1) and conferring chemoresistance to initially sensitive cells 213. Furthermore, EVs can transfer oncoproteins from drug-resistant to drug-sensitive cells. For example, in NPC, EVs rich in EGFR promote EGFR overexpression and the downregulation of intracellular ROS levels via the PI3K/AKT pathway, promoting the metastatic potential of poorly metastatic NPC cells 214. In addition, EVs produced by the K562 CML cell line contain the BCR-ABL oncoprotein, which can support the survival of BaF3 cells under IL-3 deprivation, protecting them from cell cycle arrest and apoptosis 215.

Resistance to drugs is transmitted by exchanging anti-apoptotic proteins via EVs, such as survivin. Survivin functions by inhibiting caspases, thus preventing cell death, and it is often highly expressed in various types of cancers 216. Notably, survivin is identified in EVs derived from PCa, CC, and BCs 217-219, and its presence is associated with unfavorable clinical outcomes 216. Exosomal survivin, for instance, has been demonstrated to enhance the survival of PCa cells under conditions of serum starvation and impairs the effectiveness of paclitaxel (PTX) treatment 220.

In cancer therapy, antibodies targeting PD-1/PD-L1 have demonstrated effectiveness 221. However, anti-PD-1 therapy encounters challenges due to its low response rates and PD-L1-mediated immune evasion mechanisms. Research has indicated that metastatic MeL cells release EVs that express PD-L1, and this expression can be amplified by IFN-γ, leading to the inhibition of CD8**^+^** T cell function and the promotion of cancer cell proliferation. In patients with metastatic MeL, the levels of circulating exosomal PD-L1 are positively associated with IFN-γ levels and exhibit changes during anti-PD-1 therapy, suggesting that exosomal PD-L1 may serve as a predictive marker for the outcomes of anti-PD-L1 treatment 29. Furthermore, the expression of PD-L1 on immune cells like monocytes and NK cells contributes to the deactivation of cytotoxic T-cell responses 4, 222.

Additionally, studies indicate that exosomes derived from drug-resistant cancer cells have enriched in annexin A6 protein, which inhibits the ubiquitination and degradation of EGFR. This induction of resistance to GeM is observed in triple-negative BC. In CLL, ncRNA hY4 from CLL-derived EVs can induce the expression of PD-L1 on monocytes through a TLR7-dependent mechanism 223. Inhibiting TLR signaling can counteract the upregulation of PD-L1 induced by CLL-derived EVs and may offer a potential therapeutic strategy against resistance 4. These studies emphasize the role of proteins within EVs from cancer cells in propagating resistance to anticancer drugs.

### 5.4 Resistance due to EV-mediated mRNAs and DNA

EVs containing mRNAs and DNA have emerged as active regulators of cancer progression (**Table 3**). For instance, exosomes derived from AML cells carry both VEGF and VEGF receptor (VEGFR) mRNA, promoting proangiogenic activity in HUVECs by enhancing glycolysis. This results in vascular remodeling and the development of chemoresistance 224. In another study, exosomes containing ZEB1 mRNA, a key transcription factor in the EMT, are transferred from chemoresistant NSCLC cells to chemosensitive cells, offering a new mechanism for inducing chemoresistance 225. Furthermore, research has shown that gefitinib-resistant (GR) cells exhibit higher glycerol kinase 5 (GK5) mRNA levels than GR-sensitive cells. Exosomal GK5 mRNA plays a role in mediating gefitinib resistance, and silencing GK5 can restore drug sensitivity 226. These findings suggest that mRNAs carried by EVs can be transferred to recipient cells, where they are translated into proteins, contributing to the acquisition of chemoresistance. Additionally, EVs containing mitochondrial DNA (mtDNA) have been associated with therapy-induced transition of cancer stem-like cells from dormant to activated, resulting in resistance to hormonal therapy through an oxidative phosphorylation-dependent mechanism 227.

### 5.5 Resistance due to drug efflux pumps in EVs

Tumor cells can exploit EVs to transmit chemoresistance through the horizontal transfer of drug efflux pumps (**Table 3**). Many instances of MDR in cancer therapy are associated with increased drug efflux pump expression 227, 228. Members of the ATP-binding protein family typically regulate drug efflux across the plasma membrane. These proteins use ATP to pump drugs out of the cytoplasm, preventing their accumulation in cancer cells 229, 230. Emerging evidence suggests that EVs harbor a variety of drug efflux pumps, including transporters belonging to the ATP-binding cassette (ABC) superfamily. Notable examples include ABC transporter G2 (ABCG2/BCRP, BC-resistant protein), ABC transporter C1 (ABCC1/MRP1, MDR-associated protein 1), ABC transporter B1 (P-gp/ABCB1/MDR1, MDR protein 1), and ABCA3. These pumps within EVs directly contribute to the active removal of drugs 215. Furthermore, it is proposed that these drug efflux pumps can be transferred from drug-resistant cancer cells to drug-sensitive ones through EVs, ultimately acquiring drug resistance 26, 231. ABCB1 (P-gp) is the most extensively studied and associated with resistance to multiple chemotherapeutic drugs among the ABC transporter family. Mechanistically, P-gp is enclosed within the vesicular membrane, which is then transferred to recipient cells and exposed on their surface 26. For instance, when P-gp-carrying EVs from resistant BC cells are exposed to sensitive cells, they transfer resistance to cytotoxic drugs like docetaxel and doxorubicin *in vitro* 231, 232. Notably, EVs containing P-gp from OVC and PCa cells confer taxane resistance to drug-sensitive cancer cells 233. Similarly, the acquisition of docetaxel resistance in recipient cells by transferring P-gp via EVs from docetaxel-resistant PCa cells has also been documented 234. In addition to P-gp, other members of the ABC transporter family, including ABCC1, ABCA3, and ABCG2, have been implicated in horizontal transfer mediated by EVs, contributing to drug resistance in AML 196, 235, 236 and laryngeal cells 236, respectively 196, 235, 236. Remarkably, the persistence of this intercellular transfer of drug resistance extends beyond the half-life of the drug efflux pumps themselves. Consequently, while the transfer of ABC transporters via EVs can provide partial insights into the development of drug resistance in previously sensitive cells, additional mechanisms facilitating EV-mediated intercellular transfer of drug resistance probably exist. These mechanisms might involve the regulation of these pumps at the protein level within recipient cells, offering a more comprehensive understanding of the phenomenon 237. In addition to pumping drugs, EVs also play a role in sequestering drugs, limiting their intracellular concentration and potentially leading to sublethal drug levels within the cells 238-244. For instance, EVs derived from MCF7 BC cells and Balm3arege-B-cell-lymphoma cells accumulate DOX and release it into the surrounding media, reducing its intracellular concentration 245.

Similarly, BC cells have demonstrated the ability to expel the DOX into the extracellular environment *via* vesicle formation 246. Notably, ABC transporters have been identified on the membranes of EV-like structures, aiding in drug sequestration 242. This phenomenon has been observed in regions of cell-to-cell contact between adjacent cells in mitoxantrone-resistant human BC cell lines that express ABCG2, a transporter capable of sequestering mitoxantrone 242. Additionally, EVs have been found to accumulate other ABCG2 substrates, such as imidazoacridinones and methotrexate, further underscoring the role of ABCG2-overexpressing EVs in MDR 240. Interestingly, EVs can also facilitate the removal of drugs from the extracellular space. For instance, they can reduce the extracellular levels of anti-cancer therapeutic antibodies by presenting specific antigens on their surfaces. B-cell lymphoma-derived EVs, for example, carry the CD-20 receptor, enabling them to bind to the anti-CD20 chimeric antibody rituximab and shield target cells from its effects 27. A similar phenomenon is observed in epithelial cell adhesion molecule (EpCam)-positive BC cells treated with the EpCam-specific antibody C215, suggesting a potential connection between EV release and tumor progression 247. Although some studies have explored drug sequestration by EVs using cancer cell models, the evidence from *in vivo* models and patients remains limited. Thus, further research is necessary to unveil the mechanisms underpinning drug sequestration by EVs released by cancer cells and to ascertain the clinical significance of this process in cancer patients.

### 5.6 Resistance due to CASC-released EVs

Cancer associated stromal cells (CASCs) residing within the TME also releases EVs, facilitating the transfer of drug resistance capabilities (**Table 4**).

#### 5.6.1 Involvement of CAFs released EVs

EVs derived from CAFs contain many molecules, including long lncRNAs, miRNAs, proteins, and circRNAs, and they play a significant role in conferring drug resistance to recipient cancer cells. CAFs containing lncRNA H19 induce resistance in CRC cells to oxaliplatin by activating the β-catenin pathway 248. Similarly, CAFs harboring lncRNA CCAL promote resistance of CRC cells to oxaliplatin and 5-FU by activating the Wnt/β-catenin signaling pathway 249. miRNAs are also transferred from CAFs to cancer cells via EVs, contributing to chemotherapy resistance. For instance, miR-21 present in EVs released by CAFs induces paclitaxel chemoresistance in OVC cells by downregulating the expression of apoptotic peptidase activating factor (APAF1) mRNA 250. MiR-21 is also involved in CAF EV-induced GeM resistance in PC 251. CAFs secrete EVs containing miR-92a-3p, which, upon transfer to CRC cells, activates the Wnt/β-catenin pathway and inhibits mitochondrial apoptosis, thereby promoting resistance to combined oxaliplatin and 5-FU treatment 252. CAFs also transfer miR-522 to GC cells, inhibiting ferroptosis by regulating arachidonate lipoxygenase 15 (ALOX15) expression and lipid-ROS levels, ultimately suppressing cell death and promoting resistance to cisplatin and paclitaxel 253. Additionally, CAFs-derived EVs induce resistance in NSCLC cells to cisplatin through the intercellular transfer of miRNA-130a, which is packaged into EVs with the assistance of pumilio homolog 2 (PUM2), an RNA-binding protein 254.

Furthermore, miR-20a is enriched in CAF-derived exosomes and targets the PTEN/PI3K/Akt pathway to promote cisplatin resistance in NSCLC 255. CAFs containing miR-196a within the cargo of EVs confer cisplatin resistance in head and neck squamous cell carcinoma (HNSCC) by targeting p27 and ING5, key regulators of cell cycle and apoptosis 256. CAF-derived EVs also contain miR-106b, critical in gemcitabine resistance by directly targeting TP53INP1 in PC 257. Moreover, EVs containing miR-93-5p released by CAFs confer resistance by downregulating FOXA1 and upregulating TGFβ3 258. In addition to nucleic acids, CAF-derived EVs mediate drug resistance by transferring proteins and circRNAs.

For instance, CAF-derived EVs contain Annexin A6, enhancing drug resistance in GC cells to cisplatin by activating the β1 integrin-adhesion kinase (FAK)-YAP signaling pathway 259. These EVs can also carry Wnts, glycoproteins that activate the Wnt/β-catenin pathway, leading to resistance in CRC cells to oxaliplatin *in vitro* and *in vivo* 260. Furthermore, CAF-derived circN4BP2L2 binds to EIF4A3, activating the PI3K/Akt/mTOR pathway and inducing oxaliplatin resistance in CRC cells 261. These findings underscore the active role of CAFs within the TME in regulating therapy resistance through the transmission of various molecules via EVs, representing a complex and multifaceted resistance mechanism in cancer therapy.

#### 5.6.2 Involvement of MSC-released EVs

MSC-EVs also play a role in promoting tumor progression by enhancing vascularization and influencing the TME. These MSC-EVs, primarily containing miRNAs and lncRNAs, mediate therapy resistance through intercellular communication 262. MSC-EVs containing miR-222/223 can alter the cell cycle of BC cells, inducing quiescence and dormancy, compromising the response to chemotherapy 263. The presence of the lncRNA lncPSMA3-AS1 in MSC-EVs has conferred resistance to the proteasome inhibitor bortezomib in multiple myeloma (MM) cells by increasing PSMA3 expression 264. Another example involves miR-23b, found in EVs originating from bone marrow MSCs (BMSCs) and inducing resistance in BC cells to DOX 265. Furthermore, EVs derived from BMSCs mediate drug resistance in MM cells 266. These EVs carry miR-155, which promotes stemness in MM cells and upregulates drug resistance genes such as MRP1, ABCG2, and P-gp, ultimately contributing to increased drug resistance 267.

#### 5.6.3 Involvement of TAMs and CAAs released EVs

TAMs play a significant role in regulating cancer drug resistance 268. EVs derived from TAMs (TAM-EVs) containing miRNAs are implicated in drug resistance. For instance, miR-21 and miR-223 from TAM exosomes induce cisplatin resistance in GC 269 and OVC 270. Proteins from TAM-EV, such as fibronectin and chitinase 3-like-1, impact PC cells sensitivity to GeM, possibly by activation of the ERK pathway 271. miR-365 originating from TAM-EV also plays a role in GeM resistance in PC by impacting nucleotide pools and cytidine deaminase expression 150. Reports indicate that TAMs secrete the transcription factor GATA3 via EVs, which regulates macrophage polarization and contributes to cisplatin resistance in OVC cells 272. Furthermore, exosomes derived from M2 macrophages enriched with the lncRNA CRNDE reduce cisplatin sensitivity in GC cells by inhibiting PTEN expression 273. Similarly, EVs released by adipocytes are known to transfer miR-23a/b to hepatocellular carcinoma (HCC) cells, leading to resistance against 5-fluorouracil (5-FU) by targeting the VHL/HIF axis 274. Exosomes enriched with miR-21 from cancer-associated adipocytes (CAAs) are transferred to OVC cells, promoting chemotherapy resistance through regulating APAF1 250. Additionally, CAAs from MM cells contain LOC606724 and SNHG1, which protect against apoptotic damage induced by chemotherapeutic drugs, ultimately contributing to therapy resistance 275. Moreover, adipocyte-derived exosomal MTTP inhibits ferroptosis and promotes oxaliplatin resistance in CRC through the MTTP/PRAP1/ZEB1 axis 276.

#### 5.6.4 Involvement of MDSCs and Treg-released EVs

MDSC-derived exosomes (MDSCs-Exos) are involved in various aspects of cancer, including immunosuppression, tumor angiogenesis, metastasis, resistance to immunotherapy, and drug resistance 277, 278. MeL-derived EVs induce the conversion of monocytes into monocytic MDSCs through miRNA-mediated transcriptional regulation. This process involves miR-146a, miR-100, miR-125b, and miR-155, which are enriched in the plasma of multiple myeloma (MM) patients and are closely associated with the clinical efficacy of immune checkpoint inhibitors (ICIs) 279. Moreover, exosomal miR-126a released from MDSCs induced by DOX treatment can confer resistance to BC cells against DOX therapy and promote lung metastasis 278. While the relationship between Treg-EVs and chemotherapy resistance though not fully characterized, recent research has provided insights into this aspect. CRC cells secrete exosomal miR-208b, which is transferred to recipient T cells. In these T cells, miR-208b targets programmed cell death factor 4 (PDCD4), leading to Treg expansion associated with oxaliplatin resistance and tumor growth promotion 280.

Similarly, EndCs within the TME can also play a role in contributing to drug resistance 281. Antiangiogenesis therapies (AATs) have demonstrated effectiveness against various malignancies, but the subsequent emergence of cancer vasculogenesis and disease progression often limits their efficacy. For instance, Vandetanib, a vasculogenesis inhibitor, has been shown to induce the release of exosomes enriched with VEGF. These VEGF-rich exosomes promote the formation of endothelial vessels and angiogenic mimicry in HCC. This suggests that the development of vasculogenesis and disease progression following AATs may involve communication between EndCs and cancer cells mediated by VEGF-rich exosomes 282. Furthermore, EVs carry various membrane-associated proteins with angiogenic properties. For example, EPHB2, a protein found on small EVs secreted by HNSCC cells, can stimulate cancer angiogenesis by interacting with ephrin-B2 on the surface of EndCs, activating the STAT3 signaling pathway and exacerbating resistance to anti-angiogenesis therapy 283. On the flip side, human microvascular EndCs have been reported to promote chemotherapy resistance in NPC cells through the secretion of EVs 284. These studies underscore the diverse mechanisms through which EVs derived from various non-cancer cell populations, including MSCs, TAMs, and CAAs, can contribute to therapy resistance in different cancer types by transferring molecules to cancer cells within the TME.

## 6. Role of EVs in monitoring cancer therapy response

Due to their ease of detection in various bodily fluids and unique bioactive contents, EVs have paved the way for developing novel cancer biomarkers for purposes like diagnosis, prognosis, and predicting treatment efficacy 285-288 (**Table 5**).

### 6.1 Exosomal microRNAs as a biomarker

miR-425-3p has emerged as one of the most differentially expressed miRNAs in serum exosomes of platinum-resistant patients compared to platinum-sensitive individuals. It has been linked to poor responsiveness and reduced progression-free survival (PFS) in NSCLC patients, underscoring its potential as a biomarker for predicting the response to platinum-based chemotherapy 285. Similarly, elevated levels of miR-21 in circulating exosomes are a potential biomarker in various malignancies, spanning LivC, GC, BC, CRC, OVC, and ECs. Increased exosomal miR-21 in urine is associated with urothelial carcinoma of the bladder (UCB), and PCa 289-294. Additionally, an elevated level of exosomal miR-146a-5p has demonstrated its potential as a robust predictor for cisplatin response, with lower levels indicating a higher recurrence rate in NSCLC patients 295. Furthermore, several miRNAs have been identified in serum-derived exosomes from HCC patients, with some showing positive associations with HCC progression and poor survival rates 296, 297. In contrast, others like miR-638 exhibit downregulation and negatively correlate with more aggressive tumor behavior 286, 298-301. These findings highlight the promising role of EV-derived miRNAs as cancer biomarkers, offering valuable insights into diagnosis and prognosis.

EV-associated miRNAs have shown promise as predictive indicators for assessing therapeutic responses and patient outcomes in various cancer contexts. For example, in PCa, reduced levels of miR-34a in EVs have been linked to poor survival, potentially related to how EV-miR-34a responds to DOX treatment 198. However, most EV-miRNAs in PCa appear to play a protective role. Studies have identified specific miRNAs, including hsa-let-7a-5p, hsa-miR-21-5p 302, miR-493-5p, miR-323a-3p, miR-411-5p, miR-494-3p, miR-379-5p, miR-654-3p, miR-409-3p, miR-543, and miR-200c-3p, which, when present at higher levels in serum EVs, are associated with a more favorable response to carbon ion radiotherapy (CIRT). Increased expression of these miRNAs predicts better therapy outcomes for patients undergoing CIRT 303. Moreover, the abundance of exosomal miR-146a-5p has been identified as a robust predictor for cisplatin response, while elevated levels of exosomal miR-425-3p indicate cisplatin resistance in LC 295, 304. In OVC, the analysis of EV-related miRNAs in plasma from patients resistant to platinum therapy has revealed several differentially expressed miRNAs in plasma EVs, including miR-181a, miR-21, miR-1908, miR-486, and miR-223. These EV-derived miRNAs hold promise as predictors of platinum resistance 305. In OVC, miR-21-3p, miR-21-5p, and miR-891-5p are enriched in EVs and contribute to resistance to carboplatin-based chemotherapy. Detecting these miRNAs in EVs from biofluids may offer a means of assessing patient responses to chemotherapy, thereby providing valuable information for treatment decisions 306. Exosomal miR-155 and miR-1246 is used to distinguish trastuzumab-resistant and sensitive patients. Their upregulation is closely associated with shorter DFS in early-stage patients and PFS in metastatic patients 307. Detecting exosomal miRNA levels holds great promise for personalized treatment and post-treatment disease monitoring. miRNAs like let-7 and miR-497, considered tumor suppressors, have been linked to better outcomes in response to chemotherapy or radiotherapy 308. A study by Svedman *et al.* revealed that levels of let-7g-5p and miR-497-5p increased in plasma EVs following targeted therapy in MM patients. These increases correlated with improved disease control, aligning with their roles in inhibiting tumor progression 308. Furthermore, androgen receptor splice variant 7 (AR-V7), a variant of the androgen receptor 291, has been discovered in plasma EVs and is associated with shorter PFS and overall survival (OS) in metastatic castration-resistant PCa. This finding suggests that AR-V7 in EVs may be a predictive marker for resistance to hormonal therapy in these patients 309.

### 6.2 EV-associated lncRNAs and circRNAs as biomarkers

Several deregulated lncRNAs and circRNAs found in EVs play crucial roles in cancer drug resistance and have the potential to serve as novel biomarkers. For example, a study conducted by Yang *et al.* demonstrated that circulating exosomal lncRNA UCA1 could predict the efficacy of cetuximab treatment in CRC patients. Patients with a poor response to treatment exhibited significantly higher levels of circulating exosomal UCA1 than those with a favorable response 310. In renal cancer carcinoma (RCC) patients, the expression of vesicular lncRNA HILAR was notably higher in those with metastasis than those without metastasis 311. Another study revealed a significant correlation between increased expression of EV-associated lncRNA CRNDE-h and regional lymph node metastasis and distant metastasis in CRC patients 287. Furthermore, elevated levels of vesicular lncRNA-SOX2OT were associated with shorter overall survival rates in NSCLC patients 312. Zinc finger antisense 1 (ZFAS1), which belongs to competing endogenous lncRNAs, was enriched in GC patients' serum EVs. High levels of vesicular ZFAS1 may be linked to a higher risk of lymphatic metastasis in GC patients 313. Another study demonstrated that circ_0008928 contributes to cisplatin resistance in NSCLC by regulating the miR-488/HK2 axis. The levels of circ_0008928 were upregulated in serum exosomes of cisplatin-resistant patients 314. Similarly, the upregulated expression of circPRMT5, a circRNA specific to UCB, was more likely to occur in patients with lymph node metastasis than in those without metastasis in EVs derived from serum and urine 315. Despite their potential value, detecting exosomal RNAs in early-stage cancers is challenging due to their low expression levels. A nanoparticle-based biochips method has been developed to capture circulating EVs without isolation. It enables the visualization and amplification of encapsulated RNAs *in situ* in a single step, making it advantageous for early cancer detection 316.

### 6.3 EV-associated proteins as biomarkers

EV proteins hold great promise as biomarkers for assessing cancer therapy effectiveness. For example, Glypican-1 (GPC1) is overexpressed explicitly in EVs derived from tumors. Detecting serum-derived EVs containing GPC1 can differentiate between healthy individuals, patients with benign PC, and those with early- and late-stage PC, offering high specificity and sensitivity 317. Similarly, contactin-1 is found at elevated levels in plasma EVs from MeL patients compared to those from healthy individuals, suggesting that detecting these differently expressed proteins in MeL cancer-derived EVs could play a crucial role in tumor diagnosis and monitoring 288. Studies on EV proteins in BC have also revealed interesting insights. For instance, the transfer of transient receptor potential channel 5 (TRPC5) via EVs stimulates the production of the p-gp, contributing to chemoresistance in non-resistant cells. Increased TRPC5 expression in plasma EVs predicts low chemotherapy response and a strong association with the development of acquired chemoresistance before tumor progression 213, 318. Moreover, overexpression of annexin A6 is linked to a poor response to GeM-based chemotherapy 319. Emerging evidence suggests that exosomal PD-L1 plays a role in immunosuppression and may be a potential predictor for anti-PD-1 therapy in MeL and NSCLC. High levels of EV-PD-L1 may indicate T cell exhaustion before treatment, and increased PD-L1 in EVs may predict improved anti-tumor immunity following immunotherapy in responders 159, 320-322. This phenomenon was not observed in non-responder MeL patients 159. Additionally, higher expression of PD-1 and CD28 in immune cell-secreted EVs may predict a positive response to immunotherapy in metastatic MeL, offering a new source of EVs for potential biomarker discovery 323. Furthermore, researchers have demonstrated that PCa markers, such as PSA, PSMA (prostate-specific membrane antigen), and 5T4 (oncofetal glycoprotein), are differentially positive in urinary EVs from cancer patients compared to healthy volunteers. Furtehrmore, PSA, PSMA, and 5T4 were found to decrease after therapy, suggesting that PCa markers in EVs have the potential as biomarkers for monitoring patients post-therapy. However, further validation in larger cohorts is necessary 324. Studies have also explored specific proteins associated with MeL therapy and progression in EVs, including MeL chondroitin sulfate proteoglycan (MCSP), MeL cell adhesion molecule (MCAM), low-affinity nerve growth factor receptor (LNGFR), and receptor tyrosine-protein kinase (ErbB3). These proteins exhibited changes in different MeL patients during and after targeted therapy, but their precise functions in patients undergoing treatments require further investigation 325. CD31, a marker for EndC-derived EVs that regulate T-cell responses, has also been studied. Low levels of CD31**^+^** endothelial-derived EVs in responders to ICIs corresponded to an activated immune response.

In contrast, high levels may indicate endothelial-induced tumor immune escape in non-responders with NSCLC 326. In summary, EV-associated biomolecules have the potential to serve as valuable biomarkers for assessing therapy response (**Table 5**). Although the accessibility of EVs makes them appealing for biomarker discovery, clinical validation is still needed for their routine use.

## 7. Impeding EV biogenesis, trafficking, release, and recipient cell uptake; novel approach to cancer treatment

**Figure 4:**
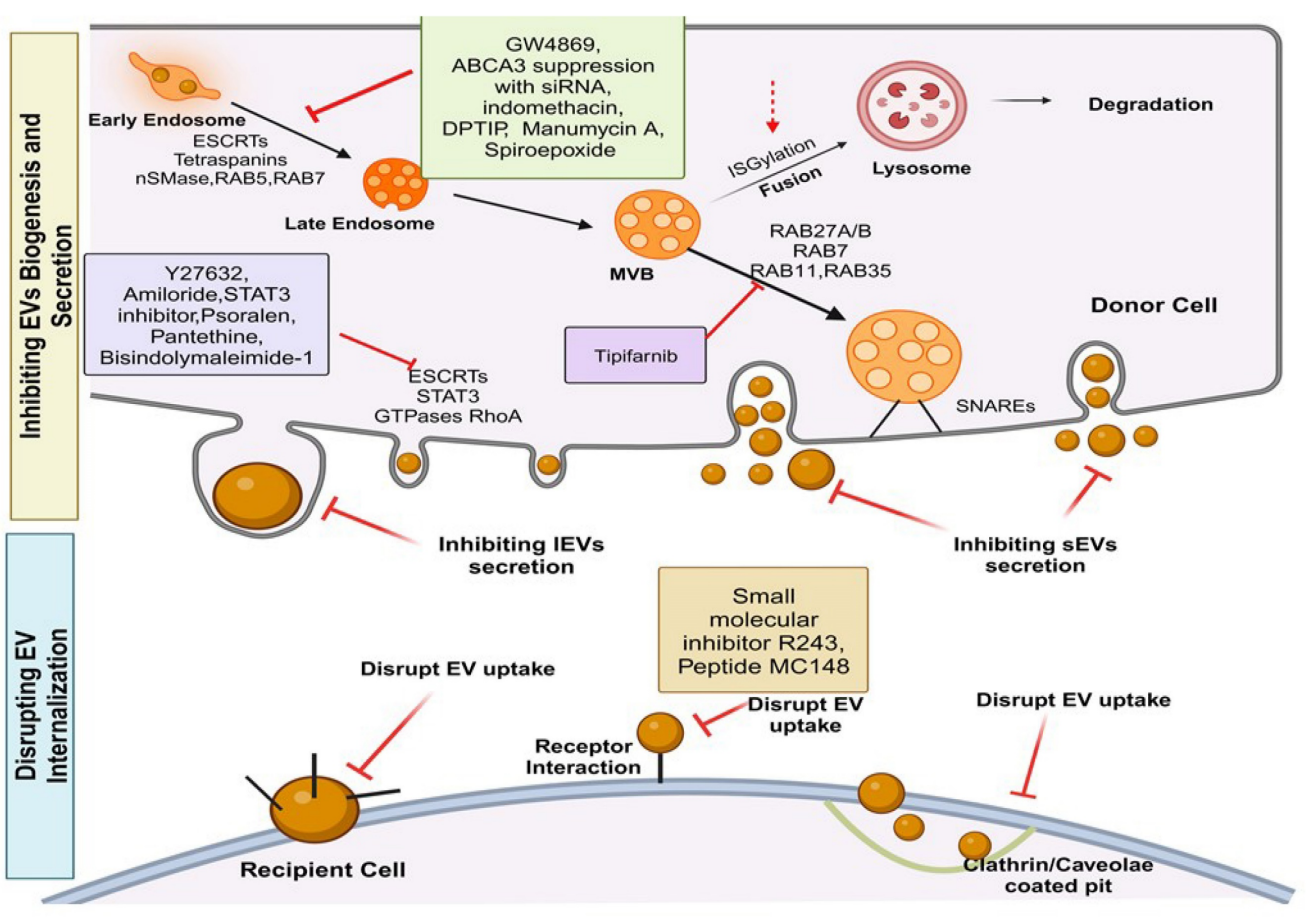
This figure illustrates strategies for overcoming drug resistance by targeting key proteins involved in the biogenesis, secretion, and uptake of EVs. Red lines represent therapeutic agents that interfere with EV biogenesis, secretion, or internalization processes, aiming to disrupt the pro-tumoral functions of EVs. Additionally, a green dotted line signifies an activator of ISGylation, a process that promotes the fusion of multivesicular bodies (MVBs) with lysosomes for degradation, thus mitigating the effects of EVs.

Recently, EVs have been utilized as a focal point for combating drug resistance. Potential strategies encompass reshaping the composition of EVs, disrupting EV-mediated interactions within the TME, impeding the processes of EV biogenesis and release, enhancing the degradation of EVs, capturing circulating EVs originating from tumors, and hindering the uptake of EVs by recipient cells 327. Additionally, investigations have delved into using exosomes derived from specific cell types and the potential engineering of EVs to serve as a delivery tool for overcoming drug resistance. Disrupting the proteins involved in EV biogenesis and secretion represents a promising avenue for cancer treatment 124, 328-330 (**Figure 4**). This includes directing attention to endosomal sorting complexes required for transport proteins (ESCRT-dependent), neutral sphingomyelinases (ESCRT-independent), tetraspanin family proteins, as well as proteins governing the trafficking and secretion of small EVs (sEVs), such as Rab family proteins, soluble N-ethylmaleimide-sensitive factor attachment protein receptors (SNAREs), Rac-1, and actin cytoskeletal proteins 329, 331, 332. Large EVs (LEVs) can potentially be inhibited by targeting small GTPase RhoA 333, membrane cholesterol 334, and the calcium-dependent enzymatic machinery responsible for the externalization of phosphatidylserine 335.

Pharmacological agents have been explored for their potential to inhibit EVs originating from tumors. For example, targeting oncoproteins like STAT3, which play a role in EV biogenesis and secretion, can be accomplished using compounds like amiloride or STAT3 inhibitors. Combining these inhibitors with chemotherapy has shown promising results, leading to increased apoptosis and reduced proliferation in chemoresistant OVC cells 336. Psoralen, a compound derived from Fructus Psoraleae, can reduce the production and release of exosomes by modulating PPAR and p53 signaling pathways, thereby overcoming resistance to DOX in BC 337. Senescent stromal cells, contributors to cancer resistance through the excessive production of EVs, can be targeted by activating SIRT1 using SRT2104, thus curbing EV production 338. Moreover, various drugs and compounds have been examined for their potential to inhibit EV biogenesis and release. These include the ROCK inhibitor Y27632, which reorganizes the cellular cytoskeleton and acts as an inhibitor of EV formation 333, 339, 340. The membrane-neutral sphingomyelinase (nSMase) inhibitor GW4869 has demonstrated the ability to block ceramide-mediated EV biogenesis 341-343, resulting in the reversal of drug resistance in OVC cells 344, and decreased tumor progression in MeL 345. D-pantethine, an inhibitor of cholesterol synthesis, has been found to reduce EV release 346, potentially due to reduced cellular cholesterol levels or the inhibition of phosphatidylserine (PS) translocation 347. Moreover, drugs manumycin A, spiroepoxide, and 2,6-Dimethoxy-4-(5-Phenyl-4-Thiophen-2-yl-1H-Imidazol-2 yl)-Phenol (DPTIP), have been shown to selectively block the release of EVs from various cancer cells by targeting neutral sphingomyelinases or ESCRT machinery 347. Additionally, the drug Bisindolylmaleimide-1 (BIM-1) has been shown to significantly inhibit EV release from BC and PCa cells 335, potentially by reducing the externalization of PS 335, 348. Tipifarnib is another compound with the potential to modulate EV biogenesis and secretion in PCa cells by inhibiting essential proteins (ALIX, nSMase2, and Rab27a) involved in EV production 349. Several other compounds, including U0126, clopidogrel, and calpeptin, have been reported to reduce EV formation and release 46.

An additional strategy to combat cancer drug resistance involves inhibiting the internalization of exosomes by target cells. For instance, MM cells internalize exosomes from BMSC, leading to reduced sensitivity to bortezomib and a chemotherapy drug. MM and BMSCs communicate through macropinocytosis and Clathrin- and caveolin-dependent endocytosis. Inhibitors of these endocytic processes in MM cells suppress the internalization of BMSC-derived exosomes, enhancing the effectiveness of bortezomib by attenuating exosome-mediated chemoresistance 350. A recent study uncovered a new way that cells take up EVs involving the interaction between a cell receptor called CCR8, the sugars on EVs' surfaces, and a molecule called CCL18. Blocking CCR8 using specific inhibitors can prevent EV uptake by GBM cells and make them more responsive to temozolomide (TMZ), a chemotherapy drug used to treat GBM 351.

Functional transfer of EVs containing MDR transporters is associated with the desensitization of cancer cells to chemotherapeutics. Therefore, reducing the proportion of these vesicular pumps could be a valuable therapeutic approach. For example, ABCA3, which promotes the secretion of EVs and the development of drug resistance, can be suppressed using siRNA or indomethacin. Inhibition of ABCA3 leads to decreased exosome production, increased intracellular drug retention, altered subcellular drug accumulation, prolonged nuclear drug retention, and enhanced sensitivity of cancer cells to drugs like DOX and pixantrone 352. Moreover, administering a proton pump inhibitor to metastatic MeL cells has improved the effectiveness of cisplatin treatment. This improvement is attributed to a reduction in the production of EVs and an increased retention of chemotherapy drugs within the tumor cells 237, 353.

Exosomal PD-L1 molecules inhibit T-cell activation in lymph nodes, contributing to cancer immune evasion. Strategies to target exosomal PD-L1 are being explored to overcome cancer resistance to immunotherapy. In one study, removing PD-L1 from cancer cell-derived exosomes inhibited cancer growth, even in models resistant to anti-PD-L1 antibodies 247. Combining anti-PD-L1 antibodies with the blockade of exosomal PD-L1 has demonstrated synergistic effects in suppressing cancer growth 247. In another study, the use of macitentan effectively inhibited the production of EV PD-L1 in BC cells. This inhibition enhanced antitumor immune responses and improved therapeutic outcomes when macitentan was used with anti-PD-L1 antibody treatment 248. Furthermore, a phototheranostic approach involving metal-phenolic networks was employed to deliver a ferroptosis inducer (Fe3+) and an exosome inhibitor (GW4869). This strategy combined photothermal therapy with exosome-based immunotherapy, reactivating T cells by counteracting exosomal PD-L1-mediated suppression and ultimately inducing robust antitumor immunity in metastatic MeL 249.

## 8. EVs as potential drug delivery vehicles

Efficient cancer treatment necessitates the precise and targeted delivery of therapeutic agents to cancer cells 354. EVs have gained attention as potential drug delivery vehicles due to their high drug-loading capacity and specific targeting capabilities, promising to overcome drug resistance in cancer treatment 29. Exosomal miRNAs have emerged as valuable tools in cancer diagnosis and therapy 355, 356. Research has demonstrated that loading EVs with miRNAs designed to target cancer-promoting genes can effectively inhibit cancer growth and reverse drug resistance. For instance, in the case of GBM, resistance to TMZ is associated with the loss of miR-151a. Introducing miR-151a into TMZ-resistant cells through exosomal delivery inhibits XRCC4-mediated DNA repair, thereby increasing sensitivity to therapy 357. Similarly, exosomal delivery of miR-122, miR-146a, and miR-567 enhances the sensitivity of HCC cells to sorafenib 358, OVC cells to docetaxel 359, and BC cells to trastuzumab 359, respectively. In addition to delivering miRNAs, EVs can carry miRNA inhibitors to achieve anti-tumor effects. For example, elevated levels of miR-374a-5p in oxaliplatin-resistant GC cells are associated with poor prognosis. A miR-374a-5p inhibitor via exosome delivery promotes cell apoptosis and overcomes drug resistance 360. Similarly, in cisplatin-resistant GC cells with high levels of miR-214, exosome-mediated delivery of a miR-214 inhibitor reverses chemoresistance and inhibits cancer growth 361. Furthermore, miR-21 induces 5-FU resistance in CRC by downregulating hMSH2 (human DNA MutS homolog 2) 362. Exosomal delivery of a miR-21 inhibitor combined with 5-FU treatment enhances the sensitivity of HER2+ cancer cells to 5-FU 363. Additionally, exosome-mediated delivery of a miR-21 inhibitor reduces the resistance of BC cells to DOX by blocking the function of oncogenic miR-21 364.

EVs can load and deliver siRNAs to tumor cells, including those within the brain, bypassing the blood-brain barrier (BBB) 365-367. For instance, exosomal delivery of siRNA efficiently silences circRNA-SORE in HCC cells. This circRNA promotes PRP19-mediated degradation of YBX1, enhancing sorafenib-induced apoptosis 368. In sorafenib-resistant cancer cells, exosomal siRNA targeting GRP78 increases the sensitivity of drug-resistant HCC cells to sorafenib 369. Notably, engineered exosomes loaded with siRNAs have been used to target PC cells and showed significant tumor inhibition in preclinical models, with clinical trials underway 370, 371. Furthermore, exosomes loaded with siRNA targeting metastasis-associated protein 1 (MTA1) enhance the therapeutic effect of GeM by inhibiting HIF-α and autophagy in luminal-b BC 372. Similarly, exosome-mediated delivery of siRNA against carnitine palmitoyltransferase 1A (CPT1A) enhances the response to oxaliplatin in CRC by inhibiting CPT1A and downstream fatty acid oxidation (FAO) 373. The delivery of si-cMet via exosomes has significantly reversed cisplatin resistance in GC 374. These findings illustrate that EV-mediated delivery of siRNAs can effectively target drug-resistant cancer cells, restoring their sensitivity to treatment. Furthermore, in the case of oxaliplatin-resistant CRC, exosomal delivery of circ-FBXW7, which binds to miR-128-3p, has demonstrated the potential to render CRC cells sensitive to oxaliplatin and inhibit drug efflux, presenting a promising therapeutic strategy 375. Additionally, the lncRNA PGM5 antisense RNA1 (PGM5AS1) has been found to inhibit acquired oxaliplatin tolerance in CRCs. The co-encapsulation of oxaliplatin and PGM5AS1 in exosomes represents an efficient approach to reversing oxaliplatin resistance 376. Furthermore, a membrane fusion technique has been developed to load macromolecular proteins into EVs for targeted delivery. In one example, researchers employed exosomal delivery of Survivin-T34A in PC, leading to increased sensitivity to GeM. Survivin-T34A, a dominant-negative mutant of survivin, triggers caspase activation and cell apoptosis by dissociating the caspase9/survivin protein complex 377.

The recombinant form of TRAIL (rTRAIL) has been extensively studied in clinical trials 378. However, the limited bioavailability of rTRAIL has posed challenges for its clinical application 379. EV-mediated delivery of TRAIL (EV-T) in a membrane-bound form has shown enhanced ability to induce cell apoptosis. Importantly, T-EV has demonstrated significant apoptotic effects in TRAIL-resistant cancer cells, suggesting that EV-TRAIL could serve as a viable alternative to rTRAIL for cancer treatment, yielding superior therapeutic outcomes 380. Furthermore, combining T-EV with other drugs, such as SCH727965 (dinaciclib), an effective CDK inhibitor, has been investigated to sensitize the cellular response pathways in cancer cells. Dinaciclib has substantially augmented the cytotoxic effects of T-EV in cancer cells expressing death receptor 5. Combining a low dose of T-EV with dinaciclib has led to a complete cancer regression in mouse models. These findings underscore the potential of combining T-EV with dinaciclib as a promising approach to enhance the effectiveness of cancer therapy 381.

## 9. EV-mediated drug delivery to overcome cancer drug resistance

Recent research has proved that EVs can serve as delivery vehicles to augment drug accumulation within tumor tissues, extend drug circulation in the bloodstream, enhance therapeutic effectiveness, and mitigate systemic toxicity 382-388. One promising avenue involves using exosomes as a platform for delivering the chemotherapeutic drug paclitaxel (PTX) to combat MDR cancer. Exosomes derived from macrophages are loaded with PTX through sonication, resulting in a formulation known as exoPTX. ExoPTX enhances PTX solubility and circumvents drug resistance mediated by P-gp. This exosome-mediated delivery approach exhibits high PTX loading and sustained release within resistant cancer cells, thereby increasing cytotoxicity compared to free PTX 419. Furthermore, EVs loaded with PTX have demonstrated effective targeting and antitumor effects in LC, BC, and PCs 383, 386, 389, 390. Notably, PTX's limited ability to traverse the BBB has been addressed through the use of PTX-loaded exosomes, improving the treatment of GBM 391. Autologous PCa cell-derived EVs have also been explored as efficient carriers for PTX, enhancing its cytotoxic effects 382. Exosomes derived from BMSCs loaded with PTX and GeM were employed to overcome chemotherapy resistance in PC. This approach demonstrated low systemic toxicity, excellent tissue penetration, and improved anticancer efficacy, offering a prospective strategy for targeted PC therapy 392. DOX is known for its effectiveness against various cancers but is limited by poor biocompatibility and cardiotoxicity. EVs have been shown to mitigate DOX's cardiotoxicity by reducing its accumulation in the heart, making it a potential means of enhancing its clinical application 393, 394. DOX-loaded EVs derived from various sources, including macrophages, DCs, HEK293 cells, and red blood cells, have exhibited superior anticancer effects compared to free DOX 385, 389, 395, 396. Furthermore, to enhance drug delivery strategies, researchers have explored the development of co-delivery systems involving EVs and inorganic nanocarriers, aiming to leverage the advantages of both approaches. The combination of EVs with hydrogels and liposomes has gained significant attention in recent studies. This synergistic approach improves the targeting capabilities of EVs and enhances drug encapsulation efficiency, surpassing the performance of standalone inorganic nanocarriers like gold nanoparticles and therapeutic nano-bioconjugates 433-434. For example, Li *et al.* have developed hybrid nanoparticles inspired by biology. These nanoparticles merge tumor exosomes expressing CD47 with cRGD-modified liposomes to co-deliver the chemotherapeutic drug triptolide (TP) and miR-497. This innovative combination exhibits potent anticancer activity through a synergistic mechanism involving inhibiting the PI3K/Akt/mTOR signaling pathway, induction of excessive ROS production, and repolarization of macrophages from an M2 to an M1 phenotype. This approach effectively overcomes chemoresistance in OVC 397. In summary, EVs serve as an effective nano delivery platform for various anticancer drugs, potentially mitigating their original limitations in clinical use.

Apart from acting as delivery tools, EVs have been engineered with functional nanomaterials to enhance their efficiency, specificity, and safety in cancer therapy 398. For instance, in the case of PTX delivery, researchers have loaded PTX into exosomes (exoPTX) and modified them with aminoethylanisamide-polyethylene glycol (AA-PEG). This modification enhances loading capacity and targeting capabilities, significantly improving the therapeutic effectiveness of PTX 383. In treating metastatic peritoneal cancer, thermo-peritoneal chemotherapy has proven effective but is hindered by drug delivery challenges and the development of resistance. Innovative approaches in drug delivery involve the genetic engineering of fibroblasts to produce exosomes expressing CD47, which are then combined with thermosensitive liposomes (referred to as gETLNPs) for the delivery of docetaxel. This novel combination therapy enhances the targeted delivery of cancer drugs and successfully overcomes drug resistance 399. Additionally, researchers have engineered EVs derived from HEK293T cells by attaching a hyaluronic acid derivative with octadecyl tails (referred to as lipHA) to the EV membrane, resulting in engineered EVs known as lipHA-engineered hEVs. These modified EVs efficiently deliver chemotherapeutic drugs to drug-resistant cancer cells through CD44-mediated targeting. Simultaneously, they inhibit drug efflux by suppressing P-gp expression, effectively reversing cancer drug resistance 400. Researchers have genetically engineered fibroblasts to produce exosomes expressing CD47, fused with thermosensitive liposomes (gETLNPs) to deliver docetaxel. This innovative combination therapy enhances cancer-targeted drug delivery and successfully overcomes drug resistance 399. Furthermore, EVs derived from HEK293T cells have been engineered by attaching a hyaluronic acid derivative with octadecyl tails (lipHA) to the EV membrane, resulting in lipHA-engineered hEVs. These engineered EVs deliver chemotherapeutic drugs to drug-resistant cancer cells through CD44-mediated targeting. Simultaneously, they inhibit drug efflux by suppressing P-gp expression, thus effectively reversing cancer drug resistance 400. In addition to drug delivery, functionalized EVs have shown promise in radiotherapy sensitization. For instance, engineered exosomes from M1 macrophages (M1Exos) is used as sensitizers. These M1Exos were modified with catalases to alleviate tumor hypoxia and loaded with an anti-PD-L1 nanobody and a DNA damage repair inhibitor. This combination leads to effective remodeling of the immunosuppressive TME, ultimately enhancing radiotherapy efficacy 401.

## 10. Concluding remarks and future perspectives

The rapid expansion of research on EVs has shed considerable light on their versatile and pivotal roles within the TME. This comprehensive review revisits the intricate biology of EVs, encompassing their composition and uptake, which are fundamental for deciphering EV-mediated intercellular communication within the complex TME between cancer cells and CASCs, delineating their involvement in metabolic reprogramming, horizontal transfer of malignant traits, and their substantial contribution to resistance against anticancer therapeutics within the intricate TME.

Central to the discourse is the pivotal role of EV constituents, spanning proteins, nucleic acids, lipids, and metabolites, in orchestrating cell-cell communication dynamics within the TME. While much emphasis has been placed on the delivery of proteins and nucleic acids following EV internalization, there is an imperative need for further investigation into the intricate mechanisms governing the sorting and export of these bioactive cargo molecules. Understanding how these EV cargoes evade degradation post-internalization and subsequently exert their functional impact is paramount. Notably, it is worth acknowledging that not all facets of EV-mediated communication rely solely on internalization. The significance of EV-cell surface signaling cannot be understated. Technological advancements have allowed for a more confident characterization of EVs, even with reduced input material. However, adherence to rigorous guidelines that substantiate the 'extracellular' and 'vesicular' nature of isolated particles remains essential.

The TME, a complex milieu encompassing cancer cells, CASCs, and non-cellular components, hinges upon EV-mediated interactions, leading to mutual education of cancer cells and CASCs. These interactions profoundly affect the existing functions of these cellular constituents and the acquisition of novel traits. For instance, EVs have demonstrated the capacity to activate fibroblasts into CAFs and fine-tune cancer cells to promote proliferation and metastasis. Conversely, certain immune cell-derived EVs have exhibited the ability to enhance anticancer activities. Furthermore, EV-mediated signaling within the TME significantly influences angiogenesis and vascular permeability, creating a hospitable environment conducive to tumor development. Intriguingly, neurons have emerged as participants in the EV-mediated crosstalk, as cancer-derived EVs can reprogram neurons into a pro-tumorigenic phenotype. EVs also carry proteinases that contribute to extracellular matrix degradation, exacerbated by the hypoxic and acidic conditions often found within the TME. These findings offer promising avenues for targeted cancer treatment, with standard EV-mediated positive feedback loops between cancer cells and TME constituents representing attractive therapeutic targets. Beyond the modulation of specific TME components, EVs play a pivotal role in reshaping the cancer-associated metabolic landscape, conferring malignant traits, and fostering resistance to various therapeutic modalities. These multifaceted processes involve EV-mediated communication across diverse cell types, underscoring the importance of comprehensive, combination therapeutic strategies. Despite the notable progress made in EV research, many challenges remain. These encompass the standardization of EV isolation protocols, the assessment of clinical efficacy, and the need to classify various EV subtypes accurately. Furthermore, there is a critical need to develop accurate and highly sensitive tools for detecting single tumor-derived EVs, along with a deeper understanding of the trafficking and uptake of tumor EVs.

In summary, the expanding body of evidence from preclinical and clinical studies underscores the immense potential of EVs as diagnostic cancer biomarkers and as carriers for targeted drug delivery. However, several scientific challenges persist, including refining EV-based drug delivery strategies and large-scale purification techniques. To fully harness the potential of EVs in cancer therapeutics, concerted efforts from researchers, clinicians, and regulatory authorities are imperative. The ongoing progress in EV research undoubtedly holds the promise of translating laboratory findings into clinically viable tools for cancer management within the intricate TME.

## Figures and Tables

**Table 1:** Role of Tumor derived EVs in Cancer Angiogenesis

EVs Cargo	Cancer type	Molecular Mechanisms of action		Ref.
miR-9	MeL	Activation of JAK/ STAT pathway	Downregulation of suppressor of cytokine signalling 5 (SOCS5) levels	89
miR-221-3P	CC	Inhibition of Thrombospondin-2 expression	----	49
miR-21-5p	CRC	Activation of β-catenin signaling pathway, upregulation of VEGFA	Downregulation of Krev interaction trapped protein 1	87
miR-210	HCC	upregulation of STAT6	Downregulation of SMAD4	80, 98
miR-144	NPC	upregulation of VEGFA and HIF-1α	Downregulation of FBXW7	82
LncRNA-HOTAIR	GBM	upregulation of VEGF-A	----	83
LncRNA-H19	LiC	upregulation of VEGF	----	402
miRNA-25- 3p	CRC	----	Down regulation of KLF2, KLF4	86
miRNA-103	HCC	----	Down regulation of Cadherin, p120-catenin, zonula occludens-1	102

**Abbreviations**: MeLC, Melanoma cancer; CC, Cervical cancer; CRC, Colorectal cancer; HCC, Hepatocellular carcinoma; NPC, Nasopharyngeal carcinoma; GBM, Glioblastoma; LiC, Liver cancer; CRC, Colorectal cancer; SOCS5, suppressor of cytokine signalling 5; VEGFA, Vascular endothelial growth factor A; STAT, Signal transducer and activator of transcription.

**Table 2:** Exosomal miRNAs in various cancers

Cancer type	Exosomal miRNA	Expression pattern	Molecular Pathways & targets	Observation	Ref.
NSCLC	miR-3180-3p	Down	FOXP4, Flotillin-1	Inhibits the proliferation and metastasis	170
	miR-770	(-)	MAP3K1, Arginase-1, iNOS, IL-10, TGF-β, E/N-cadherin, Vimentin	Decreases invasion	171
	miR-338-3p	Down	MAPK, ERK5, CHL1, MEK4, JNK, p38	Suppresses metastasis	172.
	miR-3157-3p	Up	KLF2, TSG101, TIMP, VEGF, Occludin, CD63, ZO-1, Claudin-5, MMP-2/9	Enhances angiogenesis vascular permeability and metastasis	173
	miR-1260b	Up	PARP, Caspase-3, HIPK2	Inhibiting HIPK2 could promote tumor metastasis.	174
	miR-196a-5p, miR-155	(-)	TNF-α, IRF4, IRF5, Arg-1, TSG101, E-cadherin, Vimentin, RASSF4	Enhance the metastasis	175
GC	miR-590-5p	Down	CD63, CD9	Considered as a diagnostic marker for GC.	176
	miR-122-5p	Down	HSP70, GIT1, Twist1, TSG101, E-cadherin	Inhibits the tumorigenicity	177
	miR-10b-5p	Up	AKT, PTEN, TGFβR1, S6, KLF11	Mediate communication between GC cells and fibroblasts.	178
CRC	miR-21-5p	Up	GM130, KRIT1, TSG101	Induces angiogenesis and vascular permeability.	87
	miR-22-3p	Down	PI3K, CD63, AKT, RAP2B, CD9, HSP70	Suppresses CRC proliferation and invasion	403
	miR-10a	Down	IL-6/8, IL-1β	Exosomal-miR-10a derived from CRC cells could decrease the migration of lung fibroblasts, and levels of IL-6, IL-8, and IL-1β.	404
HCC	miR-638	(-)	ZO-1, Snail, E/N-cadherin, VE-cadherin	Considered as a prognostic marker of HCC.	181
	miR-15a	(-)	SALL4, TSG101, HLA-DR, PCNA, MMP-2/9, Caspase-3	ImpedeS HCC progression.	182
CC	miR-1468-5p	Up	TSG101, PD-1, PD-L1, HSP70, IFN-γ, HMBOX1, STAT3, SOCS1/2/3, JAK2	Immunosuppressive reprogramming of lymphatic vessels could accelerate tumor immune escape	184
	miR-663b	Up	Vinculin, TSG101, CD81, CD63	Increase angiogenesis in vascular endothelial cells	185
BC	miR-138-5p	(-)	TNF-α, IL-6, IL-1β, KDM6B,	Regulates the polarization of tumor-associated macrophages.	405
	miR-500a-5p	(-)	α-SMA, USP28, FSP1, FAP, E/N-cadherin, Vimentin, FN1, ZEB1, Snail, Slug	Increases BC cell proliferation and metastasis	406
	miR-21, miR-106b, miR-1246, miR-373, miR-96, miR-17-5p, and miR-10b		PTEN, PI3K, mapsin, PDCD4, HODX10, TBX5, DYRK1A, Syndecan-1, CCNG2, CD44, HBP1, TCF, LEF, ErbB2, FUT6, and Wnt/β-Catenin	Promotes invasion and migration	407-417
	miR-10a, miR-564, miR-217, miR-34c, miR-1226-3p, miR-21, miR-148b-3p, miR-19a-3p, miR-19b, miR-1486-3p, miR-100, miR-503, miR-17/20, and miR-148a		Akt, GNA12, GYS1, SRF, PIK/MAPK, mTOR, GIT1, KLF5, FZD8, Wnt-β-Catenin, AQP5, FOSL1, mucin1, TRIM29, CCND2/CCND3, E2F1, IL-8, and CCND1	Suppresses invasion and migration	418-425
	miR-155 and miR-132	Down	VHL/HIF, RAS, and VEGF	Promotes angiogenesis	426, 427
	miR-16, miR-503, and miR-100	(-)	VEGF, FGF2, VEGFA, mtor, and HIF-1α	Suppresses angiogenesis	428-430
	miR-7641	(-)	CD9, CD63	Enhances BC progression and metastasis	431
	miR-18b	(-)	α-SMA, β-Catenin, MMP-3/9, E/N-cadherin, Snail, Vimentin, Zeb1/2, Slug, ICAM-1	Enhances BC invasion and metastasis.	432

**Abbreviations**: NSCLC, Non-Small Cell Lung Carcinoma; GC, Gastric Cancer; CRC, Colorectal cancer; HCC, Hepatocellular carcinoma; CC, Cervical cancer; BC, Breast cancer.

**Table 3:** List of T-EVs cargos and their cancer drug resistance mechanisms

Cargo type	EV cargos	Cancer type	Targets	Drug resistant to	Outcome	Ref.
miRNAs	miR-221 & miR-222	BC	p27 and ERα	Tamoxifen	Induced drug resistance in sensitive cells	188
	miR-210	PC	mTOR signaling pathway	Gemcitabine	Activate mTOR signaling pathway	189
	miR-31-5P	RCC	MutL homolog 1 (MLH1)	Sorafenib	Downregulate MLH1 expression	191
	miR-500a-3p	GC	FBXW7	Cisplatin	Enhance stemness properties and resistance	190
	miR-222-3P	NSCLC	SOCS3	Gemcitabine	Enhance the proliferation, gemcitabine resistance, migration, invasion, and anti-anoikis of sensitive cells	192
	miR-761	SS	TRIP6, LMNA, SIRT3	Pazopanib	Confer increased resistance	193
	miR-46146	CRC	PDCD10	Oxaliplatin	Contribute to the chemoresistance transfer	433
	miR-21	CRCs	PDCD4	5-FU	Downregulate TPM1 and PTEN; promote proliferation and invasion	195
		OSCC	PTEN and PDCD4	Cisplatin	Decrease the DNA damage repair signaling	194
	miR-19b & miR-20a	ALL	---	Daunorubicin	Transfer resistance from chemoresistant cells to sensitive cells	196
	miR-208a	LC	p21	Radiotherapy	Promote cell proliferation and induce RR	200
	miR-603	GBM	IGF1 and IGF1R	Radiotherapy	Promote the CSC state and upregulate DNA repair to promote acquired resistance	201.
	miR-195-5p, miR-203a-3p, miR-9-5p,	BC	ONECUT2	Docetaxel & Doxorubicin	Promote BC stemness	197
	miR-21-5p, miR-1246, miR-1229-5p & miR-96-5p	CRC	---	Oxaliplatin, 5FU	Confer increased resistance	199
lncRNAs	SNHG14	BC	Bcl-2/Bax	Trastuzumab	Transfer resistance from chemoresistant cells to sensitive cells	203
	RP11-838N2.4	NSCLC	---	Erlotinib	Contribute to the chemoresistance transfer	204
	Linc-VLDLR	EC	ABCG2	Sorafenib, DOX & Camptothecin	Promote cell viability and cell cycle progression	207
	Linc-ROR	HCC	---	Sorafenib, DOX, & Camptothecin	Reduce chemotherapy-induced cell death	205
	H19	NSCLC	---	Gefitinib	Reduce gefitinib-induced cell cytotoxicity	208
		NSCLC	miR-615-3p/ATG7 axis	Erlotinib	Facilitate erlotinib resistance	209
		BC	---	DOX	Promote cell viability, colony-forming ability, and reduce apoptosis	210
	Lnc-SOX2	NSCLC	miR-627-3p/Smads signaling pathway	EGFR-TKI	Enhance EGFR-TKI resistance	211
Proteins	p-STAT3	CRC	----	5-FU	Contribute to acquired 5-FU resistance	212
	TrpC5	BC	P-glycoprotein	DOX	Stimulate P-gp production	213
	survivin	PC	----	PTX, ERK inhibitor & Cloroquine	Promotes cancer cell survival and therapy resistance	220
	PD-L1	---	----	Anti-PD1 Ab	Suppress function of CD8 T cells	29
	Annexin A6	BC	EGFR	Gemcitabin	Inhibit ubiquitination and degradation of EGFR and induce GeM resistance	319
mRNAs	VEGF & VEGFR	Leukaemia	VEGFR/ glycolysis pathway	Ara-C	Induce glycolysis in HUVECs and lead to vascular remodelling	224
	ZEB1	NSCLC	----	Cisplatin and gemcitabine	Induce a mesenchymal phenotype in recipient cells	225
	GK5	LC	SREBP1/SCD1 signaling pathway	Gefitinib-resistant	Inhibit mitochondrial damage, caspase activation, cell cycle arrest, and apoptosis	226
DNA	mtDNA	BC	Estrogen receptor -independent (OXPHOS)	Hormone	Promote an exit from dormancy of therapy-induced cancer stem-like cells and lead to endocrine therapy resistance	227
Drug efflux pumps	ABCB1 (P-gp)	BC	----	Docetaxel and Doxorubicin	Confers drug resistance transfer	231, 232
		OVC & PCa	----	taxane	Confers drug resistance in the drug sensitive cancer cells	233
		PCa	----	Docetaxel	Promotes drug resistance transfer	234
	ABCA3	AML	Promotes Drug Export Genes Expression and ROS Inhibition	Idarubicin	Induces expression of drug efflux pump and mediate acquired chemoresistance	235
	ABCG2	laryngeal cancer	----	Cisplatin	Induces drug resistance	236

**Abbreviations**: BC, Breast cancer; PC, Pancreatic cancer; RCC, Renal cell carcinoma; GC, Gastric cancer; NSCLC, Non-small cell lung cancer; CRC, Colorectal cancer; OSCC, Oral squamous cell carcinoma; LC, Lung cancer; GBM, Glioblastoma; CRC, Colorectal cancer; EC, Esophageal cancer; HCC, Hepatocellular carcinoma; LC, Lung cancer; OVC, Ovarian cancer; PCa, Prostate cancer; AML, Acute myeloid leukaemia; MLH1,MutL homolog 1; 5-FU, 5-fluorouracil; PDCD4, programmed cell death factor 4; DOX, doxorubicin; ABCG2, ATP-binding cassette transporter G2; PTX, Paclitaxel; GeM, gemcitabine; HUVECs, Human umbilical vein endothelial cells.

**Table 4:** List of EVs cargos from stromal cells in the TME with recipient cancer cells and resistance mechanisms identified.

Donor TME cell type	EVs cargo	Recipient cancer cell type	Drugs resistance to	Resistance mechanism identified	Ref.
CAFs	lncRNA H19	CRC	Oxaliplatin	Activation of the β-catenin pathway	248
	lncRNA CCAL	CRC	Oxaliplatin	Activation of the Wnt/β-catenin signaling pathway	249
	miR-21	OVC	Paclitaxel	Downregulation of APAF1	250
		PC	Gemcitabine	---	251
	miR-92a-3p	CRC	Oxaliplatin & 5FU	Activatation of Wnt/β-catenin pathway and inhibits apoptosis	252
	miR-522	GC	Cisplatin and Paclitaxel	Targeting ALOX15 and lipid-ROS	253
	miRNA-130a	NSCLC	Cisplatin	----	254
	miR-20a	NSCLC	Cisplatin	Targeting PTEN/PI3K/Akt pathway	255
	miR-196a	HNSCC	Cisplatin	Targeting CDKN1B and ING5	256
	miR-106b	PC	Gemcitabine	Targeting TP53INP1	257
	miR-93-5p	CRC	Radioresistance	Downregulation of FOXA1 and upregulation of TGFB3	258
	Annexin A6	GC	Cisplatin	Activation of β1 integrin-FAK-YAP signaling	259
	Wnt	CRC	Oxaliplatin	Activation of Wnt/β-catenin pathway	260
	cricN4BP2L2	CRC	Oxaliplatin	Activation of PI3K/Akt/mTOR pathway	261
MSCs	miR-222/223	BC	Carboplatin	Promotion of quiescence	263
	lncPSMA3-AS1	MM	Bortezomib	Increasing PSMA3 expression	264
	miR23b	BC	Docetaxel	Decreased MARCKS expression	265
	miR-155	MM	---	Upregulation of MRP1, ABCG2, and P-gp	267
TAMs	miR-21	GC	Cisplatin	PI3K/Akt signaling enhancement	269
	miR-223	OVC	Cisplatin	PTEN-PI3K/Akt signaling activation	270
	Fibronectin; chitinase 3-like-1	PC	Gemcitabine	Activation of the ERK signaling	271
	miR-365	PC	Gemcitabine	Upregulation of triphospho-nucleotide and inducing the expression of enzyme cytidine deaminase	150
	GATA3	OVC	Cisplatin	Induces polarization of macrophages	272
	LncRNA CRNDE	GC	Cisplatin	Inhibition of PTEN expression	273
CAAs	miR-23a/b	HCC	5-FU	Confers resistance by targeting the VHL/HIF axis	274
	miR-21	OVC	---	Regulating APAF1	250
	MTTP	CRC	Oxaliplatin	Suppresses Ferroptosis and activation of MTTP/PRAP1/ZEB1 axis	276
MDSCs & Treg	miR-126a	BC	DOX	---	278
	miR-208b	CRC	Oxaliplatin	Targeting PDCD4 to promote Treg expansion	280

**Abbreviations**: CRC, Colorectal cancer; GC, Gastric cancer; NSCLC, Non-small cell lung cancer; HNSCC, Head and neck squamous cell carcinoma; PC, Pancreatic cancer; BC, Breast cancer; MM, Multiple myeloma; OVC, Ovarian cancer; HCC, Hepatocellular carcinoma;PDCD4, programmed cell death factor 4;P-gp, P-glycoprotein; PDCD4, programmed cell death factor 4; DOX, Doxorubicin; ABCG2, ATP-binding cassette transporter G2; CAF, cancer-associated fibroblasts; MSC, Mesenchymal stem cell; TAM, Tumor associated macrophages; CAA, cancer associated adipocytes; MDSCs Myeloid derived suppressor cells; Treg, T-regulatory cells; ; 5-FU, 5-fluorouracil.

**Table 5:** EV Associated Biomarkers for predicting prognosis and therapeutic response

Cargo type	Potential EV biomarkers	Cancer Type	Therapy involved	Change	Potential application	Ref.
miRNAs	miR-425-3p	NSCLC	Chemotherapy	Upregulation	Predicting Low responsiveness	285
	miR-146a-5p	NSCLC	Chemotherapy	Downregulation	Predicting recurrence	295
	miR-638	HCC		Downregulation	Predicting poor prognosis	286
	miR-34a	PCa	Chemotherapy	Downregulation	Indicating therapy failure	198
	hsa-let-7a-5p; hsa-miR-21-5p	PCa	Radiotherapy	Upregulation	Elevated radiation response	302
	9 miRNAs	PCa	Radiotherapy	Upregulation	Predicting better therapy efficacy	434
	miR-181a, miR-21, miR-1908, miR-486, & miR-223	OVC	Chemotherapy	Upregulation	Predicting primary platinum resistance	305
	miR-21-3p, miR-21-5p, miR-891-5p	OVC	Chemotherapy	Upregulation	Correlate with the risk of OVC relapse	306
	let-7g-5p; miR-497-5p	MeL	Targeted therapy	Upregulation	Correlated with better disease control	308
mRNA	AR-V7 mRNA	PCa	Hormonal therapy	NA	Predicting therapy resistance	309
lncRNA	UCA1	CRC	Chemotherapy	Upregulation	Predicting drug resistance	310
	HILAR	RCC	---	Upregulation	Correlated with metastasis	311
	CRNDE-h	CRC	---	Upregulation	Correlated with metastasis	287
	SOX2OT	NSCLC	---	Upregulation	Predicting prognosis	312
	ZFAS1	GC	---	Upregulation	Correlate with higher metastatic risk	313
circRNAs	circ_0008928	NSCLC	Chemotherapy	Upregulation	Predicting drug resistance	314
	circPRMT5	UCB	---	Upregulation	Correlated with metastasis	315
Proteins	Glypican 1	PC	---	Upregulation	Diagnosis and monitoring	317
	Contactin 1	MeL	---	Upregulation	Diagnosis	288
	TRPC5	BC	chemotherapy	Upregulation	Predicting chemoresistance	318
	Annexin A6	BC	chemotherapy	Upregulation	Predicting poor response	319
	PD-L1	MeL	Immunotherapy	Upregulation	Predicting treatment responses	159
	PD-1 and CD28	MeL	Immunotherapy	Upregulation	Predicting good treatment responses	323
	PSA, PSMA, 5T4	PCa	Hormonal therapy	Downregulation	Monitoring treatment-related responses	324
	MCSP, MCAM, LNGFR, ErbB3	MeL	Targeted therapy	NA	Monitoring treatment-related responses	325

**Abbreviations**: NSCLC, Non-small cell lung cancer; HCC, Hepatocellular carcinoma; PCa, Prostate cancer; OVC, Ovarian cancer; MeLC, Melanoma cancer; CRC, Colorectal cancer; RCC, Renal cell carcinoma; GC, Gastric cancer; UCB, Urothelial Carcinoma of the Bladder; PC, Pancreatic cancer; BC, Breast cancer.

## Abbreviations

AML: Acute myeloid leukemia
BMSCs: Bone marrow MSCs
CAAs: Cancer-associated adipocytes
CAS: Cancer-associated stromal cells
CLL: Chronic lymphocytic leukemia
CC: Cervical cancer
CRC: Colorectal cancer
EndCs: Endothelial cells
EV: Extracellular vesicles
EC: Esophageal cancer
EMT: Epithelial-mesenchymal transition
ECM: Extracellular matrix
FGF: Fibroblast growth factors
GBM: Glioblastoma
GC: Gastric cancer
HCC: Hepatocellular carcinoma
HSP: Heat-shock proteins
ICIs: Immune checkpoint inhibitors
HNSCC: Head and neck squamous cell carcinoma
LC: Lung cancer
MeL: Melanoma
MDSCs: Myeloid-derived suppressor cells
MSCs: Mesenchymal stem cells
MHC: Major histocompatibility complex
NSCLC: Non-small cell lung cancer
NPC: Nasopharyngeal carcinoma
OVC: Ovarian cancer
PDGF: Platelet-derived growth factors
PC: Pancreatic cancer
PCa: Prostate cancer
RCC: Renal cell carcinoma
SS: Synovial Sarcoma
TME: Tumor microenvironment
TIME: Tumor immune microenvironment
TAMs: Tumor-associated macrophages
TGF-β: Transforming growth factor-beta
UCB: Urothelial carcinoma of the bladder
VEGF: Vascular endothelial growth factor
